# Membrane Permeability and Aqueous Stability Study of Linear and Cyclic Diarylheptanoids from *Corylus maxima*

**DOI:** 10.3390/pharmaceutics14061250

**Published:** 2022-06-12

**Authors:** Csenge Anna Felegyi-Tóth, Zsófia Tóth, Zsófia Garádi, Imre Boldizsár, Andrea Nagyné Nedves, Alexandra Simon, Kristóf Felegyi, Ágnes Alberti, Eszter Riethmüller

**Affiliations:** 1 Department of Pharmacognosy, Semmelweis University, 1085 Budapest, Hungary; toth.csenge@semmelweis-univ.hu (C.A.F.-T.); tothzsofi0720@gmail.com (Z.T.); garadi.zsofia@pharma.semmelweis-univ.hu (Z.G.); boldizsar.imre@pharma.semmelweis-univ.hu (I.B.); nagyne.nedves_andrea@pharma.semmelweis-univ.hu (A.N.N.); simon.alexandra@pharma.semmelweis-univ.hu (A.S.); felegyi.kristof@pharma.semmelweis-univ.hu (K.F.); alberti.agnes@pharma.semmelweis-univ.hu (Á.A.); 2 Department of Plant Anatomy, Eötvös Loránd University, 1117 Budapest, Hungary

**Keywords:** absorption, blood–brain barrier, giffonin, alnusone, hazel, mass spectrometry, PAMPA

## Abstract

Seven diarylheptanoids were isolated from *Corylus maxima* by flash chromatography and semipreparative high-performance liquid chromatography (HPLC) and identified by Orbitrap^®^ mass spectrometry (MS) and nuclear magnetic resonance (NMR) spectroscopy as linear diarylheptanoids: hirsutanonol-5-*O*-β-D-glucopyranoside (**1**), platyphyllonol-5-*O*-β-D-xylopyranoside (**4**), platyphyllenone (**5**); and cyclic derivatives: alnusonol-11-*O*-β-D-glucopyranoside (**6**), alnusone (**7**), giffonin F (**8**), carpinontriol B (**9**). Cyclic diarylheptanoids are reported in *C. maxima* for the first time. The aqueous stability of the isolated compounds and other characteristic constituents of *C. maxima*, oregonin (**2**), hirsutenone (**3**), quercitrin (**10**) and myricitrin (**11**) was evaluated at pH 1.2, 6.8 and 7.4. The passive diffusion of the constituents across biological membranes was investigated by parallel artificial membrane permeability assay for the gastrointestinal tract (PAMPA-GI) and the blood–brain barrier (PAMPA-BBB) methods. The cyclic diarylheptanoid aglycones and quercitrin were stable at all investigated pH values, while a pH-dependent degradation of the other compounds was observed. A validated ultrahigh-performance liquid chromatography-diode-array detection (UHPLC-DAD) method was utilized for the determination of compound concentrations. The structures of the degradation products were characterized by UHPLC-Orbitrap^®^ MS. Platyphyllenone and alnusone possessed log *P_e_* values greater than −5.0 and −6.0 in the PAMPA-GI and PAMPA-BBB studies, respectively, indicating their ability to cross the membranes via passive diffusion. However, only alnusone can be considered to have both good aqueous stability and satisfactory membrane penetration ability.

## 1. Introduction

Compounds of plant origin play an important role in therapeutic drug discovery by providing a large chemical diversity and covering an alternative chemical space compared with synthetic derivatives. In recent years, there has been an upsurge of interest in diarylheptanoids, a group of plant-derived phenolic compounds bearing a 1,7-diphenylheptan skeleton. The firstly discovered diarylheptanoid, namely curcumin, was isolated from the traditional herbal remedy and dietary spice, turmeric (*Curcuma longa* L.) two hundred years ago [1]. Ever since, hundreds of new compounds with 1,7-diphenylheptan structures have been identified in various plants with beneficial biological effects [2]. For instance, in our previous studies, we reported the presence of antioxidant diarylheptanoids in hazel species (common hazel, *Corylus avellana* L.; Turkish hazel, *C. colurna* L.; and the filbert, *C. maxima* Mill.) for the first time. The *C. avellana* leaves have been used in traditional medicine to treat various diseases, such as phlebitis, varicose veins and hemorrhoidal symptoms, while the leaves of *C. maxima* have been applied in traditional medicine internally, as a decoction for eczema, and externally, for treating swelling and rash [3,4,5]. These results were followed by numerous research works on *C. avellana* and the isolation of different cyclic diarylheptanoid derivatives with promising biological effects, such as antioxidant activity, antiproliferative, antimicrobial, antifungal, anthelmintic, neuroprotective and α-glucosidase inhibitory effects [6,7].

However, the “dark side” of diarylheptanoids has also come under the spotlight recently: numerous studies provided evidence about curcumin’s poor pharmacokinetic properties [8]. Its absorption was negligible in many clinical trials [8]. In addition, curcumin and some other natural diarylheptanoids such as hirsutenone, oregonin and platyphylloside are highly unstable in aqueous media at neutral and alkaline conditions [8,9,10,11].

Accordingly, studies dealing with plant-derived diarylheptanoids must not leave the physicochemical and pharmacokinetic points of view out of consideration, as that might be the Achilles’ heel of this group of highly promising natural compounds.

Therefore, the aim of this study was, on the one hand, the isolation of diarylheptanoid derivatives from *Corylus maxima* leaves and their structure elucidation by Orbitrap^®^ mass spectrometry (Orbitrap^®^ MS) and nuclear magnetic resonance (NMR) spectroscopy, considering the promising compounds isolated from *C. avellana* [6] and the fact that in the case of *C. maxima*, only a tentative characterization of the diarylheptanoids by high-performance liquid chromatography hyphenated with tandem mass spectrometry (HPLC-ESI-MS/MS) is available [5]. On the other hand, we aimed to investigate the aqueous stability of the isolated diarylheptanoids at physiologically relevant pH values, as well as the determination of their ability for transcellular passive diffusion across biological membranes by the parallel artificial membrane permeability assay for the gastrointestinal tract and for the blood–brain barrier (PAMPA-GI and PAMPA-BBB) methods.

## 2. Materials and Methods

### 2.1. Plant Material

The leaves of *Corylus maxima* Mill. were collected in Pesthidegkút, Hungary [47.572938, 18.958063] in August, 2018. Fifty 50 g samples were collected from three trees after flowering stage. Plant samples were authenticated at the Department of Pharmacognosy, Semmelweis University, Budapest, where a voucher specimen is deposited.

### 2.2. Solvents and Chemicals

HPLC grade acetic and formic acid, myricetin-3-*O*-rhamnoside, quercetin-3-*O*-rhamnoside, hirsutenone, oregonin, caffeine and rutin standards, dimethyl sulfoxide-*d*_6_ (99.8 atom% D with 0.03 vol.% TMS) and methanol-*d*_4_ (99.8 atom% D), phosphatidylcholine, cholesterol and the porcine polar brain lipid extract were purchased from Merck (Darmstadt, Germany). Ethyl acetate, *n*-hexane and methanol of reagent grade, HPLC-MS grade acetonitrile, methanol, *n*-dodecane, dimethyl sulfoxide, NaCl, HCl, Na_2_HPO_4_·7H_2_O and NaH_2_PO_4_·H_2_O were obtained from Reanal-Ker (Budapest, Hungary). HPLC-grade water was prepared with a Millipore Direct Q5 water purification system (Bedford, MA, USA). All aqueous eluents for HPLC were filtered through MF-Millipore membrane filters (0.45 μm, mixed cellulose esters) (Billerica, MA, USA) and degassed in an ultrasonic bath before use.

### 2.3. Extraction and Sample Preparation

The dried and milled plant sample (150 g) was extracted with 1500 mL of ethyl acetate, which was followed by extraction with 1500 mL of methanol in an ultrasonic bath at room temperature for 8-8 h. The extracts were evaporated to dryness under reduced pressure in a rotary evaporator at 50 °C. The dried extracts (34.8 g) were dissolved in HPLC-grade methanol and filtered through Phenex-RC 15 mm, 0.2 μm syringe filters (Gen-Lab Ltd., Budapest, Hungary).

### 2.4. UHPLC-DAD-Orbitrap^®^ MS Analyses

The *Corylus* samples were analyzed by a Waters Acquity UHPLC system equipped with a diode array detector (Waters Corporation, Milford, MA, USA) using an Acquity BEH C18 column (2.1 × 100 mm; 1.7 µm, 25 °C) (Waters Corporation) with 0.1% formic acid in water as eluent A and acetonitrile as eluent B with a flow rate of 0.3 mL/min. For the chromatographic investigation of the extracts and flash chromatography fractions, different gradient profiles were applied, while aqueous stability and PAMPA solutions were analyzed with the following gradient: from 95:5 to 0:100 *v*/*v* in 10 min. The injection volume was 10 μL. UV spectra and chromatograms were recorded at 200–400 nm and the chromatograms acquired at the UV absorption maxima of each compound (281 nm for compounds **1**, **2** and **3**, 278 nm for compounds **4** and **5**, 297 nm for compounds **6** and **7**, 257 nm for compound **8**, 248 nm for compound **9**, 255 nm for compound **10** and 261 nm for compound **11**) were used for the data evaluation.

The UHPLC-DAD method, utilized for the analysis of the samples acquired in the PAMPA experiments and for the determination of aqueous stability was validated for linearity, precision and accuracy. Linearity was determined by analyzing the isolated compounds or standards (**1**, **2**, **3**, **4**, **5**, **6**, **7** and **11**) at five concentrations (0.1, 0.5, 1, 50, 100 μM in DMSO), each in triplicate. The slope, intercept and correlation coefficient were determined by a least-squares weighted regression analysis. The method provided linear responses (r^2^ > 0.999) for all standards within the investigated range (Table S1). The LOD parameter was determined at a 3/1 and the LOQ at a 10/1 signal-to-noise ratio (Table S1). The retention time repeatability was checked with six successive runs of the samples and was found to be suitable for all compounds: the relative standard deviation was <0.15% (*n* = 6). Quality control samples were prepared at three different concentrations (low, mid and high) for each standard solution, each in triplicate. These were used to determine both the intraday and interday precision and accuracy for each standard solution, which were applied for the system suitability tests: intraday and interday precision was <10% for all the analytes, while accuracy ranged from 93.69% to 116.82% (*n* = 9) (Table S2). Blank samples (pure solvents) were analyzed to exclude any coelution of impurities with the analytes.

For obtaining high resolution mass spectrometric data, a Dionex Ultimate 3000 UHPLC system (3000RS diode array detector, TCC-3000RS column thermostat, HPG-3400RS pump, SRD-3400 solvent rack degasser, WPS-3000TRS autosampler) was used hyphenated with an Orbitrap Q Exactive Focus Mass Spectrometer equipped with an electrospray ionization source (Thermo Fischer Scientific, Waltham, MA, USA). The same chromatographic method was applied as described above. The electrospray ionization source was operated in negative ionization mode, and operation parameters were optimized automatically using the built-in software. The working parameters were as follows: spray voltage, 2500 V; capillary temperature, 320 °C; sheath gas (N_2_), 47.5 °C; auxiliary gas (N_2_), 11.25 arbitrary units; and spare gas (N_2_), 2.25 arbitrary units. The resolution of the full scan was of 70,000, and the scanning range was between 120 and 1000 *m*/*z* units. Parent ions were fragmented with a normalized collision energy of 10%, 30% and 45%.

### 2.5. Isolation Procedures

In the case of the *Corylus* ethyl acetate extract, as a starting purification step, normal-phase flash chromatography (CombiFlash Nextgen 100, Teledyne Isco, Lincoln, NE, USA) was applied using two RediSep Rf Gold^®^ 40 g HP silica columns (Teledyne Isco, USA) attached to each other and hexane as eluent A, ethyl acetate as eluent B (from 100:0 to 0:100 *v*/*v* in 20 min) with a flow rate of 40 mL/min. Four fractions were collected (*a-d*). Fractions *c* (540.3 mg) and *d* (340.1 mg) were further purified by reversed-phase flash chromatography on two RediSep Rf Gold^®^ 15.5 g reversed-phase C18 columns (Teledyne Isco, USA) using 0.1% formic acid in water as eluent A and methanol as eluent B with a flow rate of 10 mL/min (from 100:0 to 0:100 *v*/*v* in 20 min.). Fraction *c* yielded thirteen further fractions (*c*1–13). Fractions *c*3 (32.4 mg) and *c*8 (48.5 mg) were purified by a Waters 2690 HPLC system with a Waters 996 diode-array detector (Waters Corporation, Milford, MA, USA) equipped with a Luna C18 100 A reversed-phase column (150 × 10.0 mm, 5 µm; Phenomenex Inc; Torrance, CA, USA, 25 °C) using 0.1% formic acid in water as eluent A and acetonitrile as eluent B. The following gradient elution programs were applied: for fraction *c*3 from 75:25 to 70:30 *v*/*v* in 35 min and for fraction *c*8 from 65:35 to 60:40 *v*/*v* in 35 min with a flow rate of 4.6 mL/min. Compound **9** (5.2 mg) was isolated from fraction *c*3 and compound **8** (8.9 mg) from fraction *c*8.

Fraction *d* yielded six fractions (*d*1–6). Fraction *d*4 (44.3 mg) was purified by semipreparative HPLC using the same instrumentation and stationary phase as detailed above and 0.1% formic acid in water as eluent A and acetonitrile as eluent B (from 80:20 to 74:26 *v*/*v* in 30 min) with a flow rate of 4.6 mL/min. Compounds **4** (4.4 mg) and **6** (1.5 mg) were isolated from this fraction.

The methanol extract was separated by flash chromatography using the same instrumentation as above, a RediSep Rf Gold^®^ 150 g reversed-phase C18 column (Teledyne Isco, USA), 0.1% formic acid in water as eluent A and methanol as eluent B (from 95:5 to 0:100 *v*/*v* in 30 min). Altogether seven fractions were collected (*e-k*). Fractions *g* (850.4 mg) and *j* (750.7 mg) were further purified by reversed-phase flash chromatography on a RediSep Rf Gold^®^ 100 g reversed-phase C18 column (Teledyne Isco, USA) using the same eluents as above. The following gradient elution programs were applied: for fraction *g* from 70:30 to 0:100 *v*/*v* in 25 min and for fraction *j* from 90:10 to 40:60 *v*/*v* in 35 min.

Fraction *g* yielded eight further fractions (*g*1–8). Fraction *g*3 (51.0 mg) was purified by semipreparative HPLC using the same instrumentation and stationary phase as detailed above, 0.1% formic acid in water as eluent A and acetonitrile as eluent B (from 88:12 to 80:20 *v*/*v* in 30 min) with a flow rate of 4.6 mL/min to yield compound **1** (7.0 mg).

Fraction *j* yielded thirteen further fractions (*j*1–13). Fraction *j*12 (42.1 mg) was purified by semipreparative HPLC using 0.3% acetic acid in water as eluent A and methanol as eluent B (from 50:50 to 30:70 *v*/*v* in 45 min) with a flow rate of 4.6 mL/min to yield compound **5** (2.5 mg). Compound **7** (1.9 mg) was isolated from fraction *j*12 (35.2 mg) by semipreparative HPLC using 0.1% formic acid in water as eluent A and methanol as eluent B (from 70:30 to 50:50 *v*/*v* in 30 min) with a flow rate of 4.6 mL/min.

### 2.6. NMR Spectroscopy

NMR spectra were recorded in deuterated methanol (Methanol-*d*_4_) or in deuterated dimethyl sulfoxide (DMSO-*d*_6_) on a BRUKER AVANCE III HD 600 (600/150 MHz) instrument equipped with Prodigy cryoprobehead at 295 K or on a Varian DDR 600 (600/150 MHz) equipped with a 5 mm inverse-detection gradient (IDPFG) probehead, at 298 K. The pulse programs were taken from the Bruker or the Varian software library (TopSpin 3.5 or VnmrJ 3.2). Chemical shifts (*δ*) and coupling constants (*J*) are given in ppm and in Hz, respectively. ^13^C and ^1^H chemical shifts are given in ppm relative to the NMR solvent or relative to tetramethylsilane (TMS) when internal standard was used. The complete ^1^H and ^13^C assignments were achieved with widely accepted strategies based on ^1^H NMR, ^13^C NMR, DeptQ, ^1^H-^1^H COSY, ^1^H-^13^C edHSQC, ^1^H-^13^C HMBC and ^1^H-^1^H ROESY measurements.

### 2.7. Determination of Aqueous Stability

The aqueous solutions and buffers were prepared as follows: pH = 1.2, 1.0 g NaCl and 3.5 mL HCl dissolved in distilled water to achieve the final volume of 500.0 mL; pH = 6.8, 20.2 g Na_2_HPO_4_·7H_2_O and 3.4 g NaH_2_PO_4_·H_2_O dissolved in distilled water to achieve the final volume of 1000.0 mL, pH adjustment with 0.5 M NaOH or 0.5 M HCl; pH = 7.4, one PBS tablet (phosphate buffered saline, pH 7.4; Sigma Aldrich, St. Louis, MO, USA) dissolved in 200.0 mL of distilled water.

With the aforementioned buffers, 10 mM dimethyl sulfoxide stock solutions of the isolated compounds were diluted 100-fold. The initial peak area (AUC) recorded by UHPLC-DAD at the zero time point was compared to the area under the peak detected in the sample incubated for 4 h at 37 °C. Statistical differences between the AUC values were evaluated by a paired-sample t-test, changes were considered significant when the *p*-values were lower than 0.05. Compounds with no notable change in their AUC and with no degradation products detected were considered to be stable. For degradable compounds, the above-mentioned validated UHPLC-DAD method was used to examine the change in compound concentration. All experiments were performed in triplicates (*n* = 3).

### 2.8. Parallel Artificial Membrane Permeability Assay (PAMPA) Studies

The test solutions were prepared with dimethyl sulfoxide (DMSO) at the concentration of 10.0 mM. These were diluted with phosphate buffers (pH = 7.4 for the PAMPA-BBB and pH = 6.8 for the PAMPA-GI) to obtain the donor solutions (297.0 μL buffer + 3.0 μL DMSO solution) and filtered through Phenex-RC 15 mm, 0.2 μm syringe filters (Gen-Lab Ltd., Budapest, Hungary). A parallel artificial membrane permeability assay (PAMPA) system was used to determine the effective permeability (*Pe*) for the compounds of interest. Each well of the top plate (96-well polycarbonate-based filter donor plates (Multiscreen™-IP, MAIPN4510, pore size 0.45 μm; Merck, Darmstadt, Germany) was coated with 5 μL of porcine polar brain lipid extract (PBLE) solution (16.0 mg PBLE + 8.0 mg cholesterol dissolved in 600.0 μL *n*-dodecane) for the PAMPA-BBB and with 5 μL of the mixture of 8.0 mg phosphatidylcholine + 4.0 mg cholesterol dissolved in 300.0 μL *n*-dodecane for PAMPA-GI. Thereafter, 150.0 μL of the filtrate was placed on the membrane. The bottom plate (96-well PTFE acceptor plates (MultiScreen Acceptor plate, MSSACCEPTOR; Millipore, Burlington, MA, USA)), was filled with a 300.0 μL buffer solution (0.01 M PBS buffer, pH = 7.4). The donor and acceptor plates were fit, and then the sandwich system was incubated at 37 °C for 4 h in a Stat-Fax 2200. After the incubation the PAMPA plates were separated and the compound concentrations in the donor (*C_D_*(*t*)) and acceptor (*C_A_*(*t*)) solutions, as well as in the donor solution at the zero time point (*C_D_*(0)) were determined by UHPLC-DAD. The effective permeability and the membrane retention in the PAMPA-BBB (1) and the PAMPA GI (2) experiments were calculated by the following equations [12]:

(1) $$P_{e}=\frac{-2.303}{A\left(t-\tau_{SS}\right)}\cdot \left(\frac{V_{A}\cdot V_{D}}{V_{A}+V_{D}}\right)\cdot \mathrm{lg}\left[1-\left(\frac{V_{A}+V_{D}}{\left(1-\mathrm{MR}\right)\cdot V_{D}}\right)\times \left(\frac{C_{A}\left(t\right)}{C_{D}\left(0\right)}\right)\right]$$

(2) $$P_{e}=\frac{-2.303}{A\left(t-\tau_{SS)}\right)}\cdot \left(\frac{1}{1+r_{a}}\right)\cdot \mathrm{lg}\left[-r_{a}+\left(\frac{1+r_{a}}{1-\mathrm{MR}}\right)\times \left(\frac{C_{D}\left(t\right)}{C_{D}\left(0\right)}\right)\right]$$

where *Pe* is the effective permeability coefficient (cm/s), *A* is the filter area (0.24 cm^2^), *V_D_* and *V_A_* are the volumes in the donor (0.15 cm^3^) and acceptor phases (0.30 cm^3^), *t* is the incubation time (s), *τ_SS_* is the time (s) to reach steady-state (240 s), *C_D_*(*t*) is the concentration (mol/cm^3^) of the compound in the donor phase at time *t*, *C_D_*(0) is the concentration (mol/cm^3^) of the compound in the donor phase at time 0, MR is the estimated membrane retention factor (the estimated mole fraction of solute lost to the membrane) and *r_a_* is the sink asymmetry ratio (gradient-pH-induced), defined as

(3) $$r_{a}=\frac{V_{D}}{V_{A}}\times \frac{P_{e}^{\left(A\to D\right)}}{P_{e}^{\left(D\to A\right)}}$$

(4) $$\mathrm{MR}=1-\frac{C_{D}\left(t\right)}{C_{D}\left(0\right)}-\frac{V_{A}}{V_{D}}\frac{C_{A}\left(t\right)}{C_{D}\left(0\right)}$$

All experiments were performed in triplicates on three consecutive days (*n* = 9), caffeine standard was used as positive, while rutin as negative control. *C*log*P* values were calculated by ChemAxon Marvin 22.3.

## 3. Results and Discussion

### 3.1. Structure Elucidation by UHPLC-Orbitrap^®^ MS and NMR

The isolated compounds were identified by Orbitrap^®^ mass spectrometry hyphenated to UHPLC separation (Table 1) and by NMR spectroscopic methods (see the Supplementary data).

The NMR data of compound **1** ([M-H]^−^ *m*/*z* 507.1902, C_25_H_31_O_11_) indicated the presence of a diarylheptanoid skeleton and a glucose moiety. The quaternary ^13^C resonances at *δ* 143.3, 143.5, 145.2 and 143.5 ppm (C-4″, 4′, 3′, 3″) suggested that the aromatic rings were substituted with hydroxyl groups, while the resonance at *δ* 209.3 ppm (C-3) revealed the presence of a carbonyl group in the heptane chain. The set of resonances in the ^13^C spectrum at *δ* 101.9, 73.7, 77.0, 70.2, 77.1 and 61.4 ppm (C_Glc_-1, 2, 3, 4, 5, 6) were typical for the presence of a glucose moiety. The HMBC correlation between the anomeric ^13^C resonance and the ^1^H resonance at *δ* 4.04 ppm (H-5) confirmed that the glucose unit was attached to the diarylheptanoid at C-5 position. Based on this information, the compound was identified as hirsutanolol-5-*O*-β-D-glucopyranoside, a linear diarylheptanoid glycoside. The NMR data were similar to those published earlier [13].

The ^1^H NMR spectrum of compound **5** ([M-H]^−^ *m*/*z* 295.1337, C_19_H_19_O_3_) indicated the presence of two *para*-substituted aromatic rings. Two olefinic resonances appeared at *δ* 6.85 (dt, ^2^*J*_H,H_ = 15.9 Hz, ^3^*J*_H,H_ = 6.8 Hz, H-5) and 6.08 (dt, ^2^*J*_H,H_ = 15.9 Hz, ^4^*J*_H,H_ = 1.4 Hz, H-4) ppm. Furthermore, four methylene groups were identified. The ^13^C resonance at *δ* 199.5 ppm (C-3) revealed the presence of a carbonyl group. These spectroscopic data suggested a linear diarylheptanoid structure. Based on the additional 2D correlations, the unsaturation was located at the C-4–C-5 position, while the carbonyl group was located at the C-3 position. Therefore, the compound was identified as platyphyllenone. The ^1^H and ^13^C NMR resonances were identical to those published in a previous report [14].

The NMR spectra of compound **4** ([M-H]^−^ *m*/*z* 445.1870, C_24_H_29_O_8_) showed analogous resonances with compound **5** (platyphyllenone) but lacking the olefinic resonances. However, an additional series of resonances appeared at *δ* 102.7, 73.5, 76.9, 69.8 and 66.0 ppm (C_Xyl_-1, 2, 3, 4 and 5) in the ^13^C NMR spectrum indicating the presence of a xylose moiety. The HMBC correlation between the anomeric ^13^C resonance and the ^1^H resonance at *δ* 3.99 ppm (H-5) confirmed the xylose unit linked to the diarylheptane at the C-5 position. Thus, the compound was identified as platyphyllonol-5-*O*-β-D-xylopyranoside. The NMR resonances were similar to those of previously reported [15].

The aromatic NMR resonances of compound **6** ([M-H]^−^ *m*/*z* 473.1810, C_25_H_29_O_9_) confirmed the presence of a diarylheptanoid moiety, while the additional series of ^13^C resonances at *δ* 102.2, 73.7, 70.1, 76.9, 76.4 and 61.2 ppm suggested the structure was a diarylheptanoid glycoside. The ^13^C resonance at *δ* 210.5 ppm was evident for a carbonyl group. Furthermore, nine aliphatic resonances along with their HSQC crosspeaks recommended the presence of five methylene units in the heptane chain. The ^1^H resonance at *δ* 4.08 ppm and its HSQC correlation to ^13^C resonance at *δ* 74.8 ppm indicated the presence of an oxymethine function. The TOCSY spectrum confirmed that this function was located in the aliphatic chain. The 2D NMR correlations confirmed the substitution pattern of the chain: the carbonyl group was in C-9, while the oxymethine function was at the C-11 position. Based on these results, the structure of the aglycone was identified as alnusonol. The HMBC correlation between the anomeric ^1^H resonance (*δ* 4.24 ppm) and the ^13^C resonance at *δ* 74.8 ppm (C-11) revealed the sugar moiety was located at the C-11 position. On the basis of this information, the structure was established as alnusonol-11-*O*-β-D-glucopyranoside.

In the case of compound **7** ([M-H]^−^ *m*/*z* 293.1184, C_19_H_18_O_3_), the heating of the NMR sample was necessary to obtain sharper, more resolved signals. The NMR data suggested a cyclic diarylheptanoid structure. The ^1^H NMR spectrum indicated the presence of two 1,2,4-trisubstitued aromatic rings. Two olefinic resonances appeared at *δ* 6.95 (dt, ^2^*J*_H,H_ = 15.5 Hz, ^3^*J*_H,H_ = 7.5 Hz, H-11) and 6.60 (dt, ^2^*J*_H,H_ = 15.5 Hz, ^4^*J*_H,H_ = 1.2 Hz, H-10) ppm, and eight further resonances suggested the presence of four methylene groups. The ^13^C NMR spectrum showed a single carbonyl resonance at *δ* 200.6 ppm (C-9). The two-dimensional NMR spectra revealed that the unsaturation was at the C-10–C-11 position, while the carbonyl group was at the C-9 position. Based on these data, the compound was identified as alnusone. Early NMR report on alnusone [16] highlights only a few characteristic ^1^H resonances, thus, herein, we provide a more complete NMR assignment.

The NMR spectra of compound **8** ([M-H]^−^ *m*/*z* 369.1350, C_21_H_22_O_6_) were characteristic for a diaryl ether heptanoid derivative. The five aromatic resonances in the ^1^H NMR spectrum suggested the presence of a disubstituted and a pentasubstituted aromatic moiety. The ^1^H resonances at *δ* 3.99 (s, 3H, OCH_3_-2), 3.67 (s, 3H, OCH_3_-4) ppm confirmed the presence of two methoxy substituents. Based on their HMBC correlations, they were located at the C-2 and C-4 positions of the pentasubstituted ring. The resonances at *δ* 6.41 (d, ^3^*J*_H,H_ = 12.1 Hz, 1H, H-7) and 5.29 (dd, ^3^*J*_H,H_ = 12.1 Hz, ^3^*J*_H,H_ = 9.1 Hz, 1H, H-8) ppm revealed the presence of an olefinic function, while the resonance at *δ* 4.29 (t, ^3^*J*_H,H_ = 9.8 Hz, 1H, H-9) ppm suggested the occurrence of an oxymethine group in the heptane chain. The ^13^C NMR spectrum displayed a carbonyl resonance at *δ* 215.6 (C-11) ppm. Furthermore, three methylene groups were identified. Based on the COSY correlations, the olefin function and the oxymethine group were adjacent to each other: they were located at positions C-7–8 and C-9. The correlations of the HMBC spectra revealed that the carbonyl group was at the C-11 position. Thus, the structure was determined as giffonin F. The ^1^H and ^13^C NMR resonances were analogous to the literature data [17].

The ^1^H and ^13^C NMR spectrum of compound **9** ([M-H]^−^ *m*/*z* 343.1187, C_19_H_20_O_6_) suggested a cyclic diarylheptanoid structure. Three oxymethine ^1^H resonances appeared at *δ* 4.71 (dd, ^3^*J*_H,H_ = 11.9 Hz, ^3^*J*_H,H_ = 4.4 Hz, H-12), 4.22 (d, ^3^*J*_H,H_ = 10.1 Hz, H-10) and 3.87 (d, ^3^*J*_H,H_ = 10.1 Hz, H-11) ppm. The ^13^C resonance at *δ* 215.6 ppm (C-9) suggested a carbonyl group in the heptane chain. Based on the correlations of the 2D spectra, the hydroxyl groups were located at positions C-10, C-11 and C-12, while the carbonyl group was at the C-9 position. Therefore, the structure was established as carpinontriol B. The ^1^H and ^13^C NMR assignments were identical to those published earlier [18].

### 3.2. Determination of Aqueous Stability

The aqueous stability of the isolated diarylheptanoids and that of the standards oregonin (**2**), hirsutenone (**3**), quercitrin (**10**) and myricitrin (**11**) were also determined. The latter two flavonol glycosides were previously reported as the main compounds in *Corylus maxima* leaf extracts [5]. The presence of the linear diarylheptanoids **2** and **3** was also proven by spiking the extract with the standards in two different chromatographic systems. The aqueous stability was investigated at three physiological pH values: at pH 1.2 in simulated gastric fluid, at pH 6.8 in simulated intestinal fluid and at pH 7.4.

The cyclic diarylheptanoid aglycones, namely, alnusone (**7**), giffonin F (**8**), carpinontriol B (**9**) and the flavonoid quercitrin (**10**), were stable at all three investigated pH values (Table 2) with no notable change in their chromatographic peak areas and with no degradation products detected by UHPLC-DAD.

A pH-dependent degradation was observed for compounds **1**, **2**, **3**, **4**, **5**, **6** and **11**, thus, in their case, the UHPLC-DAD method was validated for linearity, precision and accuracy to be utilized for the determination of compound concentrations at the initial time point and after 4 h of incubation at 37 °C (Tables S1 and S2). Statistical differences between the concentrations at the initial time point and after treatment (incubation at different pH values) were evaluated by a paired-sample t-test. Decreases in compound concentrations were considered significant when *p* < 0.05 (results are shown in Table 2).

All these aforementioned constituents were stable at pH 1.2 and showed the highest degradation rate at pH 7.4, with the diarylheptanoid glycosides **1**, **2**, **6**, and myricitrin (**11**) being the most labile (with 63.26 ± 1.93%, 59.93 ± 2.85%, 61.76 ± 0.58% and 48.44 ± 6.15% final concentrations at pH 7.4, respectively). The concentrations of compounds **3**, **4** and **11** decreased significantly only at pH 7.4, while those of compounds **1**, **2**, **5** and **6** showed a significant decrease both at pH 6.8 and 7.4 (Table 2).

The structures of the degradation products detected in the stability studies were characterized by UHPLC-Orbitrap^®^ MS.

### 3.3. Characterization of the Degradation Products by UHPLC-Orbitrap^®^ MS

**Figure 1:**
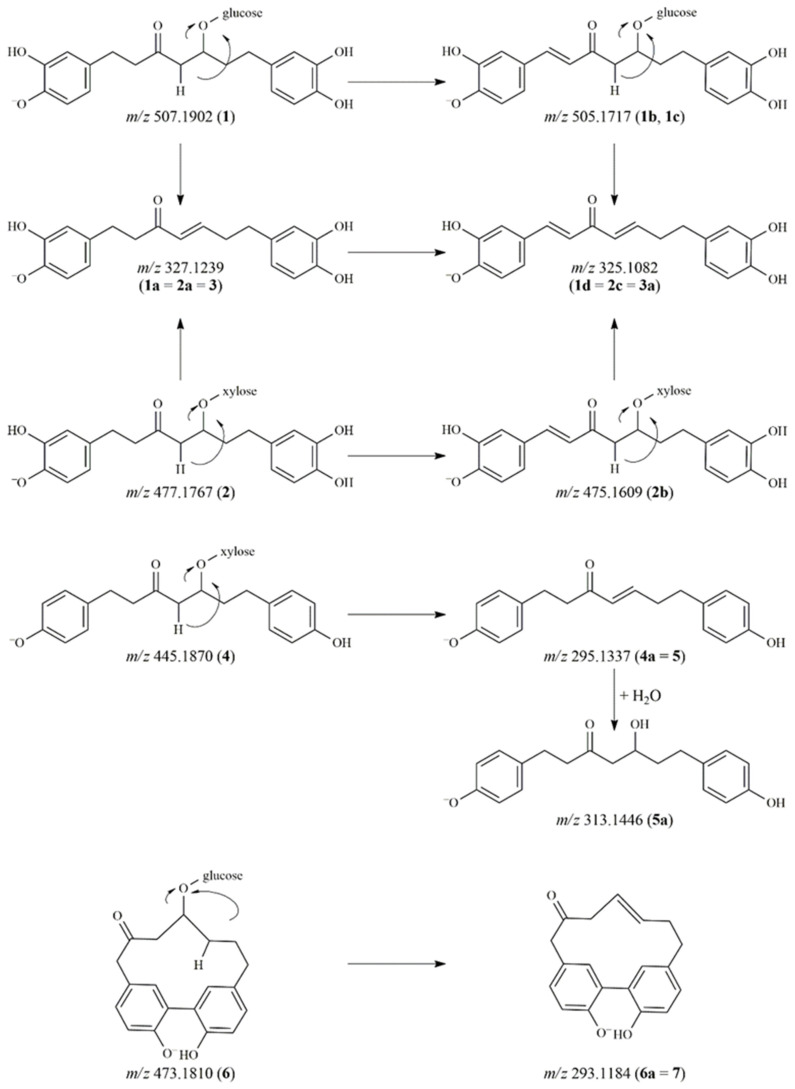
Proposed degradation pathways of diarylheptanoids.

**Figure 2:**
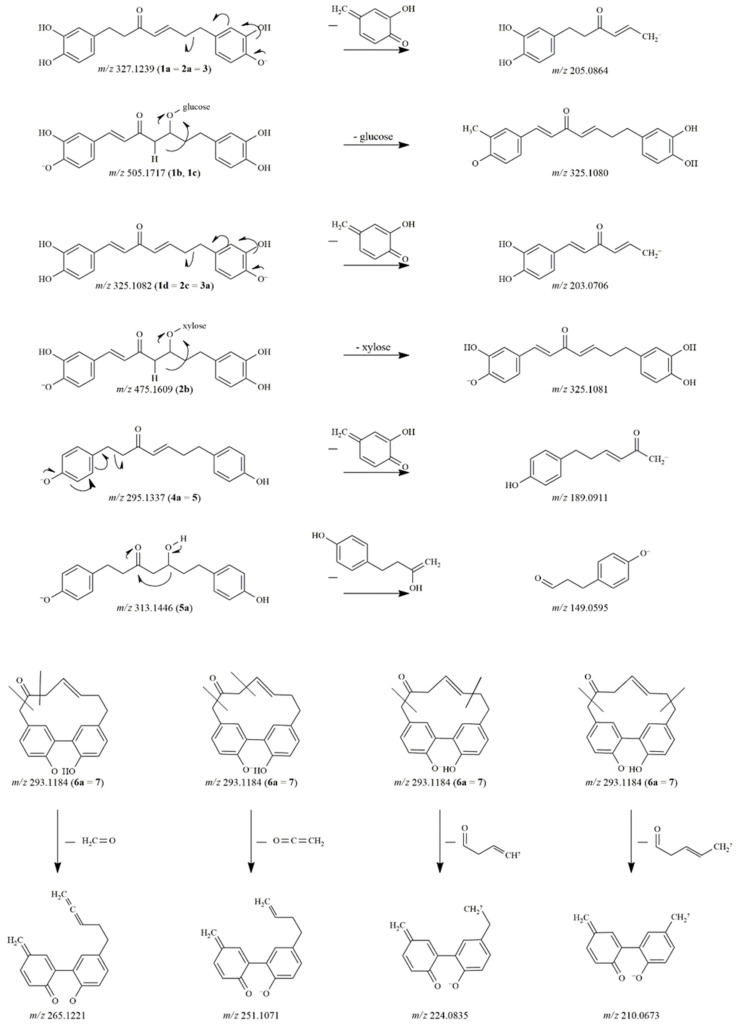
The proposed fragmentation pathways of diarylheptanoid degradation products.

The reaction mixtures of the compounds were analyzed by UHPLC-Orbitrap^®^ MS in negative ionization mode (Table 1). The proposed degradation products and the tentative characterization of the diarylheptanoid compounds are depicted in Figure 1 and Figure 2. In the case of compound **1** (hirsutanonol-5-*O*-β-D-glucopyranoside), four main degradation products were observed. One of the decomposition products was detected at *m*/*z* 327.1239 (**1a**). The difference of 180 Da between **1a** and the molecular ion of the original compound refers to the loss of a hexose moiety.

The characteristic fragment ions at *m*/*z* 205.0864 and 179.0699 indicated **1a** to be hirsutenone [3]. After comparing the retention time and the mass spectral data with those of a reference standard, **1a** was confirmed as hirsutenone. Two other degradation products at *m*/*z* 505.1718 (**1b**, **1c**) were observed in the mixture. The molecular formula was C_25_H_29_O_11_ in both cases, thus these peaks were assumed to be isomers. During their collision-induced dissociation, both **1b** and **1c** lost a sugar moiety and showed product ions at *m*/*z* 325.1080 and *m*/*z* 203.0706 in their MS/MS spectra with a difference of 2 Da compared to the fragment ions of compound **1** and the molecular and fragment ions of **1a** (*m*/*z* 327.1239 and *m*/*z* 205.0864). Accordingly, we presumed that a double bond was formed in the heptane chain during the degradation of **1**.

Additionally, the aglycone ion at *m*/*z* 325.1082 could also be found in the mixture as a degradation product (**1d**), namely 1,7-bis(3,4-dihydroxyphenyl)hepta-1,4-dien-3-one.

The degradation pattern of oregonin (**2**) was similar to that of compound **1**. The aglycone hirsutenone (**2a**) (corresponding to **1a** and **3**) as well as the derivative containing a double bond in the alkyl chain (**2b**) were detected at *m*/*z* 327.1239 and *m*/*z* 475.1609, respectively. The aglycone of **2b** was also observed at *m*/*z* 325.1083 (**2c**). The decomposed derivative of hirsutenone (**3**) was detected at *m*/*z* 325.1082 (**3a**). The instability of hirsutenone is well-known in the literature, however, its degradation products have not previously been identified [11].

The glycosidic bond of compound **4** was hydrolyzed producing the platyphyllenone aglycone (**4a**), which corresponded to the reference compound **5**. Compound **5** (*m*/*z* 295.1338) was modified to form **5a** with its molecular ion at *m*/*z* 313.1446. The difference of 18 Da refers to the addition of a water molecule. The product ions of **5a** at *m*/*z* 163.0753 and 149.0595, produced by different neutral losses, indicated the presence of a keto and a hydroxyl group on the alkyl chain [5]. Based on the fragmentation pattern and the elemental composition (C_19_H_21_O_4_), **5a** was characterized as platyphyllone.

In the case of compound **6**, only one breakdown product was detected at *m*/*z* 293.1184 (6a), where the difference was again 180 Da as compared to the molecular ion of the original compound. Thus, **6a** was identified as alnusone, the aglycone of compound **6** (alnusonol-11-*O*-β-D-glucopyranoside). This proposed structure was further confirmed when its retention time was compared to that of the reference substance alnusone (**7**). The fragment ions of **6a** (corresponding to **7**) at *m*/*z* 265.1221, 251.1071, 224.0834 and 210.0673 were generated by a rearrangement of the deprotonated molecular ion and the subsequent opening of the diarylheptanoid cycle [19].

The flavonoid myricitrin (**11**) formed two isomers at *m*/*z* 925.1686 and 925.1682 (**11a** and **11b**), and two further products at *m*/*z* 895.1583 (**11c**) and *m*/*z* 941.1633 (**11d**). Based on literature data [20], **11a** and **11b** were identified as dimers of myricitrin. Fragmentation of the dimers yielded ions at *m*/*z* 779.1099 [M-H-146]^−^ and *m*/*z* 633.0499 [M-H-146-146]^−^, corresponding to the product ions generated by the cleavage of one and two rhamnose moieties, respectively. In addition, the dimers can undergo further oxidation, resulting in the formation of **11c** and **11d** [20].

### 3.4. Parallel Artificial Membrane Permeability Assay (PAMPA) Studies

Among the investigated compounds only platyphyllenone (**5**) and alnusone (**7**) possessed log *P*_e_ values greater than −5.0 (−4.92 ± 0.07 and −4.90 ± 0.17, respectively) in the PAMPA-GI experiments, and greater than −6.0 in the PAMPA-BBB studies (−5.24 ± 0.25 and −4.66 ± 0.14, respectively) (Table 2). Accordingly, these constituents can be considered to have good membrane penetration ability [21]; thus, it might be assumed that they are absorbed in the gastrointestinal tract and cross the blood–brain barrier via passive diffusion. However, since platyphyllenone (**5**) was proven to be unstable in the aqueous media of the PAMPA models, results for this compound need to be treated with caution. However, the extent of decomposition at pH 6.8 and 7.4 (94.98 ± 2.10% and 90.40 ± 1.52% final concentrations compared to the initial value, respectively) cannot be considered significant enough to notably modify the PAMPA results.

Compounds **1**, **2**, **3**, **4**, **6**, **8**, **10** and **11** were not detected in the acceptor phase of the PAMPA models suggesting that they were unable to cross the lipid membranes of the GIT and BBB (Table 2). The cyclic diarylheptanoid aglycone, carpinontriol B (**9**) (344.1 g/mol, *c*log *P* 1.6) was detected in the acceptor phase of the PAMPA-GI model (not it the PAMPA-BBB), but the calculated log *P_e_* value (−5.49 ± 0.30) suggested a poor membrane permeability for this compound as well.

It must be pointed out that the results of the PAMPA experiments cannot be considered fully accurate in the case of the constituents proven to be chemically unstable in aqueous media at the investigated pH values (compounds **1**, **2**, **3**, **4**, **5**, **6** and **11**). Nevertheless, the diarylheptanoid and flavonol glycosides (compounds **1**, **2**, **4**, **6**, **10** and **11**) are rather unlikely to be able to cross the model membranes by passive diffusion due to their high molar mass and polarity (Mw > 440, *c*log *P* < 2.5). In addition, the maximum decomposition rate measured was 48.44%, in the case of myricitrin (**11**), thus the extent of the degradation during the PAMPA experiments would not justify the complete rejection of the results. Therefore, in the case of the glycoside derivatives, the negative results can be considered valid regardless of the chemical instability. However, further studies may be needed to investigate the membrane permeability of the labile linear diarylheptanoid aglycones, platyphyllenone (**5**) and hirsutenone (**3**), after stabilization.

In addition, it was quite interesting to observe that although neither platyphyllonol-5-*O*-β-D-xylopyranoside (**4**) nor alnusonol-11-*O*-β-D-glucopyranoside (**6**) crossed the model membranes of the PAMPA, the aglycones, platyphyllenone (**5**) and alnusone (**7**) formed from these compounds by decomposition in aqueous media both appeared in the acceptor phase.

## 4. Conclusions

In the present study, we identified and isolated seven diarylheptanoids from *Corylus maxima* for the first time: two linear diarylheptanoid glycosides hirsutanonol-5-*O*-β-D-glucopyranoside (**1**) and platyphyllonol-5-*O*-β-D-xylopyranoside (**4**), a linear aglycone: platyphyllenone (**5**), a cyclic diarylheptanoid glycoside (alnusonol-11-*O*-β-D-glucopyranoside (**6**)) and three cyclic aglycones (alnusone (**7**), giffonin F (**8**) and carpinontriol B (**9**)). The structures of the compounds were elucidated by UHPLC-Orbitrap^®^ MS and NMR spectroscopy. Cyclic diarylheptanoid derivatives were reported in *C. maxima* for the first time.

The aqueous stability of the isolated compounds at three physiologically occurring pH values (1.2, 6.8 and 7.4) as well as their passive diffusion across biological membranes (GIT and BBB) were investigated together with the characteristic constituents of *C. maxima*, oregonin (**2**), hirsutenone (**3**), quercitrin (**10**) and myricitrin (**11**).

Our results indicated that among the investigated compounds, solely the cyclic diarylheptanoid aglycone alnusone (**7**) had both a good aqueous stability and satisfactory membrane penetration ability. The other cyclic diarylheptanoid aglycones were found to be stable in aqueous environment, but the ones with a larger molar mass and higher polarity, giffonin F (**8**) and carpinontriol B (**9**), were not able to cross the lipid membranes in the PAMPA-GI and PAMPA-BBB models.

All the linear diarylheptanoids (compounds **1**, **2**, **3**, **4** and **5**) and the cyclic diarylheptanoid alnusonol-11-*O*-β-D-glucopyranoside (**6**) demonstrated significant pH-dependent decomposition in aqueous media. The linear compounds **3** and **4** were labile only at the highest investigated pH value, while **1**, **2**, **5** and the cyclic derivative **6** were significantly decomposed both at pH 6.8 and 7.4. Among the diarylheptanoid derivatives, **1**, **2** and **6** were the most unstable at pH 7.4. Based on our PAMPA results, among them, only platyphyllenone (**5**) can be considered to be able to cross the lipid membranes of the GIT and the BBB by passive diffusion.

The flavonoid glycosides quercitrin (**10**) and myricitrin (**11**) showed a poor membrane penetration ability in both the PAMPA models. In addition, myricitrin was found to be unstable in aqueous medium, its concentration significantly decreasing at pH 7.4.

The recent study might corroborate the therapeutic potential of naturally occurring cyclic diarylheptanoids in terms of physicochemical and pharmacokinetic properties in addition to their beneficial biological effects that have already been revealed: cyclic diarylheptanoid compounds have gained interest because of their remarkable in vitro anti-cancer, antioxidant, melanogenesis inhibitory [22] and neuroprotective activities [23], as well as in vivo antitumor [24], anti-inflammatory and antioxidant effects [25]. In order to be able to draw definite conclusions, a higher number of molecules should be evaluated. However, our results justify further investigations on naturally occurring cyclic diarylheptanoids as potential therapeutic agents bringing the ones with a lower molecular weight and higher lipophilicity into focus.

## Figures and Tables

**Table 1:** HR-MS data of the diarylheptanoids and flavonoid compounds, and their degradation products.

No.	[M-H]^−^ (*m*/z) Experimental	[M-H]^−^ (*m*/z) Calculated	Error (ppm)	Fragment Ions (*m*/z) (Molecular Formula, Mass Error in ppm)	Molecular Formula	Proposed Compound
**1**	507.1870	507.1866	1.76	**327.1239** (C_19_H_19_O_5_, 3.67), **205.0864** (C_12_H_13_O_3_, 2.16), 179.0699 (C_10_H_11_O_3_, −1.81), **121.0280** (C_7_H_5_O_2_, −3.48)	C_25_H_32_O_11_	hirsutanonol-5-*O*-β-D-glucopyranoside *
**1a**	327.1239	327.1233	3.69	**205.0864** (C_12_H_13_O_3_, 2.16), 179.0699 (C_10_H_11_O_3_, −1.81), **121.0280** (C_7_H_5_O_2_, 3.48)	C_19_H_20_O_5_	hirsutenone
**1b**	505.1718	505.1710	2.66	**325.1080** (C_19_H_17_O_5_, 2.93), **203.0706** (C_12_H_11_O_3_, 1.56), 161.0598 (C_10_H_9_O_2_, 0.58), 135.0435 (C_8_H_7_O_2_, −3.98), 121.0280 (C_7_H_5_O_2_, −3.48)	C_25_H_30_O_11_	1,7-bis(3,4-dihydroxyphenyl)hepta-1,4-dien-3-one-glycoside
**1c**	505.1717	505.1710	2.25	**325.1080** (C_19_H_17_O_5_, 2.93), **203.0706** (C_12_H_11_O_3_, 1.56), 161.0598 (C_10_H_9_O_2_, 0.58), 135.0434 (C_8_H_7_O_2_, −4.41), 121.0280 (C_7_H_5_O_2_, −3.48)	C_25_H_30_O_11_	1,7-bis(3,4-dihydroxyphenyl)hepta-1,4-dien-3-one-glycoside
**1d**	325.1082	325.1076	3.67	**203.0706** (C_12_H_11_O_3_, 1.56), 161.0596 (C_10_H_9_O_2_, −0.94), 135.0438 (C_8_H_7_O_2_, −2.19), 121.0280 (C_7_H_5_O_2_, −3.48)	C_19_H_18_O_5_	1,7-bis(3,4-dihydroxyphenyl)hepta-1,4-dien-3-one
**2**	477.1767	477.1761	3.85	**327.1234** (C_19_H_19_O_5_, 2.09), **205.0863** (C_12_H_13_O_3_, 1.94), 179.0699 (C_10_H_11_O_3_, −1.81), **121.0281** (C_7_H_5_O_2_, −2.91)	C_24_H_29_O_10_	oregonin *
**2a**	327.1239	327.1233	3.67	**205.0864** (C_12_H_13_O_3_, 2.16), 179.0699 (C_10_H_11_O_3_, −1.81), **121.0281** (C_7_H_5_O_2_, −2.91)	C_19_H_20_O_5_	hirsutenone
**2b**	475.1609	475.1604	2.20	**325.1081** (C_19_H_17_O_5_, 3.30), **203.0704** (C_12_H_11_O_3_, 0.51), 161.0598 (C_10_H_9_O_2_, 0.58), 135.0441 (C_8_H_7_O_2_, 0.08)	C_24_H_28_O_10_	1,7-bis(3,4-dihydroxyphenyl)hepta-1,4-dien-3-one-xyloside
**2c**	325.1083	325.1076	3.78	**203.0706** (C_12_H_11_O_3_, 1.56), 161.0597 (C_10_H_9_O_2_, 0.20), 135.0438 (C_8_H_7_O_2_, −2.06)	C_19_H_18_O_5_	1,7-bis(3,4-dihydroxyphenyl)hepta-1,4-dien-3-one
**3**	327.1239	327.1233	3.67	**205.0863** (C_12_H_13_O_3_, 1.94), 179.0699 (C_10_H_11_O_3_, −1.81), **121.0280** (C_7_H_5_O_2_, −3.48)	C_19_H_20_O_5_	hirsutenone
**3a**	325.1082	325.1076	3.57	**203.0706** (C_12_H_11_O_3_, 1.56), 161.0596 (C_10_H_9_O_2_, −0.94), 151.0385 (C_8_H_7_O_3_, −3.13), 135.0438 (C_8_H_7_O_2_, −2.06), 121.0281 (C_7_H_5_O_2_, −2.91), 109.0281 (C_6_H_5_O_2_, −2.81)	C_19_H_18_O_5_	1,7-bis(3,4-dihydroxyphenyl)hepta-1,4-dien-3-one-glycoside
**4**	445.1870	445.1862	2.82	295.1339 (C_19_H_19_O_3_, 3.32), 189.0912 (C_12_H_13_O_2_, 1.20)	C_24_H_30_O_8_	platyphyllonol-5-*O*-β-D-xylopyranoside
**4a**	295.1337	295.1334	2.91	**189.0911** (C_12_H_13_O_2_, 0.39)	C_19_H_20_O_3_	platyphyllenone
**5**	295.1338	295.1334	3.22	**189.0911** (C_12_H_13_O_2_, 0.39)	C_19_H_20_O_3_	platyphyllenone
**5a**	313.1446	313.1440	3.78	**163.0753** (C_10_H_11_O_2_, −0.42), **149.0595** (C_9_H_9_O_2_, −1.22)	C_19_H_22_O_4_	platyphyllone, platyphyllonol
**6**	473.1810	473.1812	2.68	**293.1183** (C_19_H_17_O_3_, 3.74), 265.1220 (C_18_H_17_O_2_, −0.78), 251.1070 (C_17_H_15_O_2_, 1.59), **224.0834** (C_15_H_22_O_2_, 0.79), 210.0673 (C_14_H_10_O_2_, −1.14), 197.0597 (C_13_H_9_O_2_, 0.08)	C_25_H_30_O_9_	alnusonol-11-*O*-β-D-glucopyranoside
**6a**	293.1184	293.1178	3.65	265.1221 (C_18_H_17_O_2_, −0.89), 251.1071 (C_17_H_15_O_2_, 1.71), **224.0834** (C_15_H_22_O_2_, 0.79), 210.0673 (C_14_H_10_O_2_, −1.14), 197.0596 (C_13_H_9_O_2_, −0.53)	C_19_H_18_O_3_	alnusone
**7**	293.1184	293.1178	3.85	265.1221 (C_18_H_17_O_2_, −0.89), 251.1071 (C_17_H_15_O_2_, 1.71), **224.0835** (C_15_H_22_O_2_, 1.47), 210.0673 (C_14_H_10_O_2_, −1.14), 197.0596 (C_13_H_9_O_2_, −0.53)	C_19_H_18_O_3_	alnusone
**8**	369.1350	369.1338	3.32	**339.0873** (C_19_H_15_O_6_, 2.85)	C_21_H_22_O_6_	giffonin F
**9**	343.1187	343.1182	3.11	**283.0976** (C_17_H_15_O_4_, 4.03), 269.0819 (C_16_H_13_O_4_, 3.99), 211.0756 (C_14_H_11_O_2_, 1.26)	C_19_H_20_O_6_	carpinontriol B
**10**	447.0934	447.0927	2.60	**301.0343** (C_15_H_9_O_7_, 0.18), **300.0274** (C_15_H_8_O_7_, 3.27), 271.0247 (C_14_H_7_O_6_, 3.71), 255.0296 (C_14_H_7_O_5_, 2.96)	C_21_H_20_O_11_	quercitrin *
**11**	463.0885	463.0877	3.08	**316.0223** (C_15_H_8_O_8_, 3.55), 287.0197 (C_14_H_7_O_7_, 4.10), 271.0249 (C_14_H_7_O_6_, 4.38), 242.0219 (C_13_H_6_O_5_, 2.27), 178.9976 (C_8_H_3_O_5_, 0.50)	C_21_H_20_O_12_	myricitrin *
**11a**	925.1686	925.1675	1.98	**779.1099** (C_36_H_27_O_20_, 0.45), **633.0499** (C_30_H_17_O_16_, −2.79), 435.0356 (C_22_H_11_O_10_, 0.85)	C_42_H_38_O_24_	myricitrin dimer derivative
**11b**	925.1682	925.1675	1.39	**779.1106** (C_36_H_27_O_20_, 1.32), **633.0532** (C_30_H_17_O_16_, 2.42), 597.0872 (C_28_H_21_O_15_, −1.42), 513.0441 (C_27_H_13_O_11_, −3.35), 435.0369 (C_22_H_11_O_10_, 3.85)	C_42_H_38_O_24_	myricitrin dimer derivative
**11c**	895.1583	895.1569	2.20	**749.1026** (C_35_H_25_O_19_, 4.79), 585.0311 (C_29_H_13_O_14_, 1.86), 557.0357 (C_28_H_13_O_13_, 0.14)	C_41_H_36_O_23_	myricitrin derivative
**11d**	941.1633	941.1624	1.52	**897.1686** (C_41_H_37_O_23_, −4.43), **751.1133** (C_35_H_27_O_19_, −1.81), 527.0229 (C_27_H_11_O_12_, −4.08), 393.0253 (C_20_H_9_O_9_, 1.63)	C_42_H_38_O_25_	myricitrin derivative

* Comparison with authentic standard.

**Table 2:** Results of the aqueous stability studies: compound concentration after 4 h of incubation at 37 °C compared to the initial value (%) (*n* = 3) and the PAMPA experiments’ log *P_e_* values (*n* = 9).

	Aqueous Stability			log *P_e_* PAMPA-BBB (*n* = 9)	log *P_e_* PAMPA-GI (*n* = 9)	*c*log *P*
	pH = 1.2 (*n* = 3)	pH = 6.8 (*n* = 3)	pH = 7.4 (*n* = 3)			
hirsutanonol-5-*O*-β-D-glucopyranoside (**1**)	97.03 ± 2.74	95.95 ± 1.52 *	63.26 ± 1.93 *	n.d.	n.d.	1.3
oregonin (**2**)	98.67 ± 2.43	91.22 ± 3.01 *	59.93 ± 2.85 *	n.d.	n.d.	1.9
hirsutenone (**3**)	99.63 ± 0.37	96.22 ± 2.87	83.80 ± 2.41 *	n.d.	n.d.	3.9
platyphyllonol-5-*O*-β-D-xylopyranoside (**4**)	100.47 ± 1.7	98.78 ± 0.91	89.79 ± 2.00 *	n.d.	n.d.	2.6
platyphyllenone (**5**)	99.45 ± 1.05	94.98 ± 2.10 *	90.40 ± 1.52 *	−5.24 ± 0.25	−4.92 ± 0.07	4.5
alnusonol-11-*O*-β-D-glucopyranoside (**6**)	100.92 ± 2.92	74.03 ± 1.39 *	61.76 ± 0.58 *	n.d.	n.d.	1.6
alnusone (**7**)	99.86 ± 0.50	101.55 ± 2.14	100.47 ± 1.87	−4.66 ± 0.14	−4.90 ± 0.17	4.2
giffonin F (**8**)	99.97 ± 1.01	102.68 ± 2.45	100.92 ± 2.02	n.d.	n.d.	2.8
carpinontriol B (**9**)	102.75 ± 1.09	101.51 ± 1.75	103.28 ± 1.81	n.d.	−5.49 ± 0.30	1.6
quercitrin (**10**)	100.91 ± 0.53	102.35 ± 1.85	100.75 ± 0.96	n.d.	n.d.	0.9
myricitrin (**11**)	96.23 ± 2.46	99.94 ± 0.55	48.44 ± 6.15 *	n.d.	n.d.	0.6

Abbreviations: n.d.: not detected in the acceptor phase; PAMPA-GI: parallel artificial membrane permeability assay for the gastrointestinal tract; PAMPA-BBB: parallel artificial membrane permeability assay for the blood–brain barrier. * *p* < 0.05 compared with the initial solutions.

## Data Availability

Not applicable.

## Supplementary Materials

The following supporting information can be downloaded at: https://www.mdpi.com/article/10.3390/pharmaceutics14061250/s1, Table S1. UHPLC-DAD method validation: linearity, LOD and LOQ values. Table S2. UPLC-DAD system suitability tests: precision and accuracy. Figures S1–S37: NMR spectra and spectral data of compound **1**, **4**–**9**.
