# Supplementary material for: Membrane Permeability and Aqueous Stability Study of Linear and Cyclic Diarylheptanoids from Corylus maxima
Source: Pharmaceutics. 2022 Jun 12;14(6):1250. doi: 10.3390/pharmaceutics14061250 (PMC9231376; doi:10.3390/pharmaceutics14061250)
Supplement: Supplementary file 1 [file pharmaceutics-14-01250-s001.zip › pharmaceutics-1757620-supplementary.pdf]

pharmaceutics

MDPI

Supplementary Materials: Membrane Permeability and Aqueous Stability Study of Linear and Cyclic Diarylheptanoids from *Corylus maxima*

Csenge Anna Felegyi-Tóth, Zsófia Tóth, Zsófia Garádi, Imre Boldizsár, Andrea Nagyné Nedves, Alexandra Simon, Kristóf Felegyi, Ágnes Alberti and Eszter Riethmüller

Table S1. UHPLC-DAD method validation: linearity, LOD and LOQ values.

Compound	Regression Equation	Weight Factor	R^2	Regression Range (µM)	LOD (µM)	LOQ (µM)
hirsutanonol-5-*O*-β-D-glucopyranoside (**1**)	y=2203.16x−495.38	$1/x^2$	0.9999	0.5-100	0.14	0.45
oregonin (**2**)	y=4417.70x−555.09	-	1.0000	0.5-100	0.12	0.4
hirsutenone (**3**)	y=27927.05x+1536.21	-	0.9996	0.5-100	0.04	0.13
platyphyllonol-5-*O*-β-D-xylopyranoside (**4**)	y=1073.90x+22.67	$1/x^2$	0.9994	0.5-100	0.15	0.5
platyphyllenone (**5**)	y=14650.51x−1011.18	$1/x^2$	0.9999	0.5-100	0.05	0.15
alnusonol-11-*O*-β-D-glucopyranoside (**6**)	y=6145.06−710.82	$1/x^2$	1.0000	0.5-100	0.03	0.1
alnusone (**7**)	y=6217.00x+125.05	-	1.0000	0.5-100	0.03	0.1
myricitrin (**11**)	y=20978.24x−1489.97	$1/x^2$	0.9994	0.5-100	0.02	0.05

Table S2. UPLC-DAD system suitability tests: Precision and accuracy.

Nominal conc. (µM)	Precision (RSD%)		Accuracy (%)	
	Intraday	Interday	Intraday	Interday
**hirsutanonol-5-*O*-β-D-glucopyranoside (1)**				
100	0.13	0.73	100.42	99.63
10	0.93	1.83	100.96	100.67
0.5	0.66	2.65	99.89	111.35
**oregonin (2)**				
100	0.77	0.99	100.03	99.75
10	0.96	2.03	102.15	103.43
0.5	2.56	2.84	101.96	112.56
**hirsutenone (3)**				
100	0.05	2.09	101.82	110.28
10	0.3	1.84	96.92	95.60
0.5	0.6	1.55	100.12	100.63
**platyphyllonol-5-*O*-β-D-xylopyranoside (4)**				
100	0.11	0.18	101.53	101.73
10	0.46	0.56	98.72	103.36
0.5	2.12	4.94	96.97	106.8
**platyphyllenone (5)**				
100	1.18	1.84	105.14	107.2
10	0.91	3.06	93.69	94.16
0.5	3.48	8.45	103.96	101.51
**alnusonol-11-*O*-β-D-glucopyranoside (6)**				
100	0.46	0.52	98.86	104.05
10	0.56	2.44	100.62	108.99
0.5	1.74	2.32	98.61	109.28
**alnusone (7)**				
100	0.18	0.33	100.47	100.78
10	0.19	0.42	101.20	99.17
0.5	6.69	8.74	101.78	103.14

**myricitrin (11)**				
100	0.80	1.91	100.79	103.54
10	0.49	2.14	102.56	105.61
0.5	5.71	8.24	99.01	116.82

NMR data of compound **1** (hirsutanolol-5-*O*-β-D-glucopyranoside): ^{1}H NMR (DMSO-d_6, 600 MHz, 295 K): δ 6.60 (m, 2H, H-5′, H-5″), 6.59 (m, 1H, H-2″), 6.56 (d, $^{4}J_{H,H}$=2.0 Hz, 1H, H-2′), 6.43 (dd, $^{3}J_{H,H}$=8.0 Hz, $^{4}J_{H,H}$=2.0 Hz, 1H, H-6″), 6.40 (dd, $^{3}J_{H,H}$=8.0 Hz, $^{4}J_{H,H}$=2.0 Hz, 1H, H-6′), 4.16 (d, $^{3}J_{H,H}$=7.8 Hz, 1H, H_{Glc}-1), 4.04 (m, 1H, H-5), 3.69 (dd, $^{2}J_{H,H}$=11.6 Hz, $^{3}J_{H,H}$=1.6 Hz, 1H, H-Glc-6a), 3.47 (dd, $^{2}J_{H,H}$=11.6 Hz, $^{3}J_{H,H}$=5.3 Hz, 1H, H_{Glc}-6b), 3.13 (t, $^{3}J_{H,H}$=8.6 Hz, 1H, H_{Glc}-3), 3.08 (m, 1H, H_{Glc}-5), 3.06 (m, 1H, H_{Glc}-4), 2.91 (t, $^{3}J_{H,H}$=8.6 Hz, 1H, H_{Glc}-2), 2.75 (dd, $^{2}J_{H,H}$=16.3 Hz, $^{3}J_{H,H}$=6.3 Hz, 1H, H-4a), 2.69 (m, 2H, H-2), 2.56 (m, 1H, H-4b), 2.45 (m, 1H, H-7a), 2.42 (m, 1H, H-7b), 1.68 (m, 1H, H-6a), 1.62 (m, 1H, H-6b). ^{13}C NMR (DMSO-d_6, 150 MHz, 295 K): δ 209.3 (C-3), 145.3 (C-3″), 145.2 (C-3′), 143.5 (C-4′), 143.3 (C-4″), 133.1 (C-1″), 132.1 (C-1′), 119.1 (C-6″), 118.9 (C-6′), 116.1 (C-2″), 115.9 (C-2′), 115.7 (C-5′, C-5″), 101.9 (C_{Glc}-1), 77.1 (C_{Glc}-5), 77.0 (C_{Glc}-3), 74.3 (C-5), 73.7 (C_{Glc}-2), 70.2 (C_{Glc}-4), 61.4 (C_{Glc}-6), 47.4 (C-4), 45.0 (C-2), 37.3 (C-6), 30.4 (C-7), 28.6 (C-1).

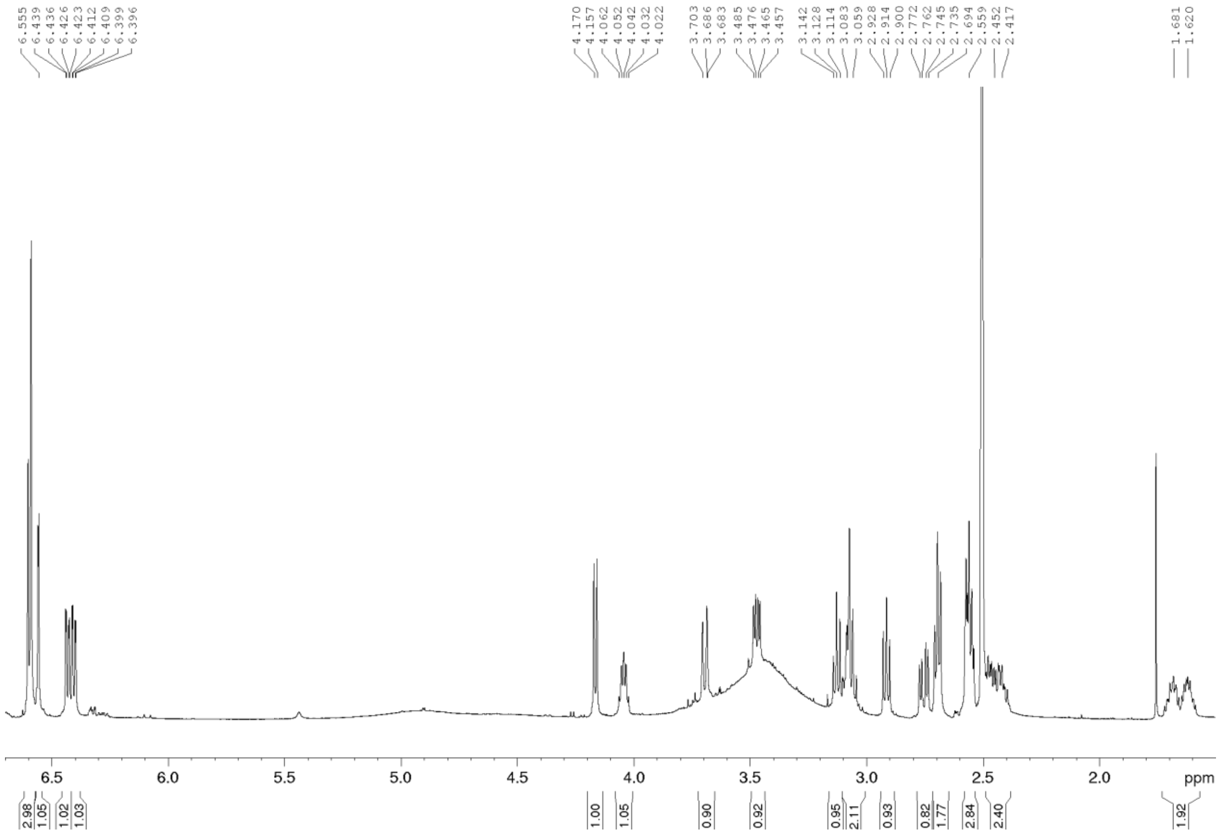

Figure S1. ^{1}H NMR spectrum of hirsutanolol-5-*O*-β-D-glucopyranoside (**1**) (DMSO-d_6, 295 K).

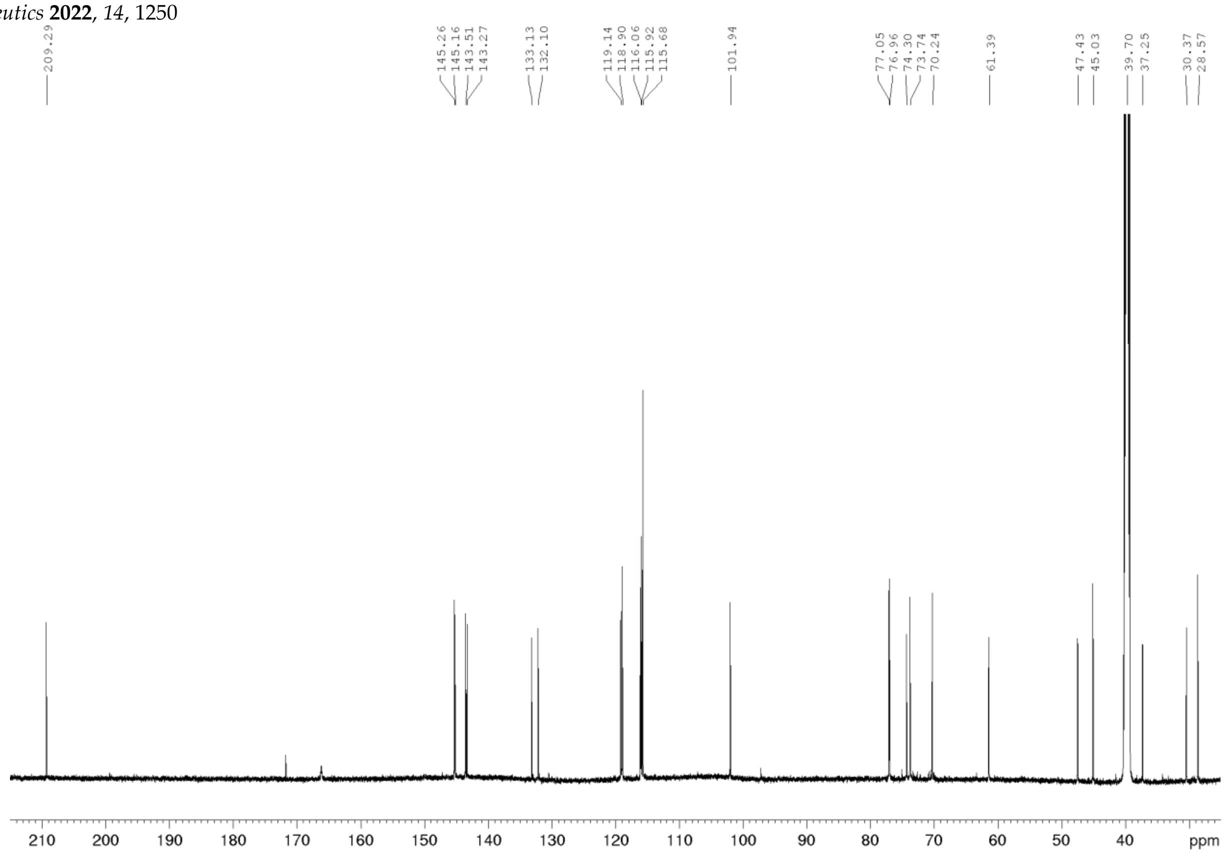

Figure S2. ^{13}C NMR spectrum hirsutanolol-5-*O*-β-D-glucopyranoside (**1**) (DMSO-d_6, 295 K).

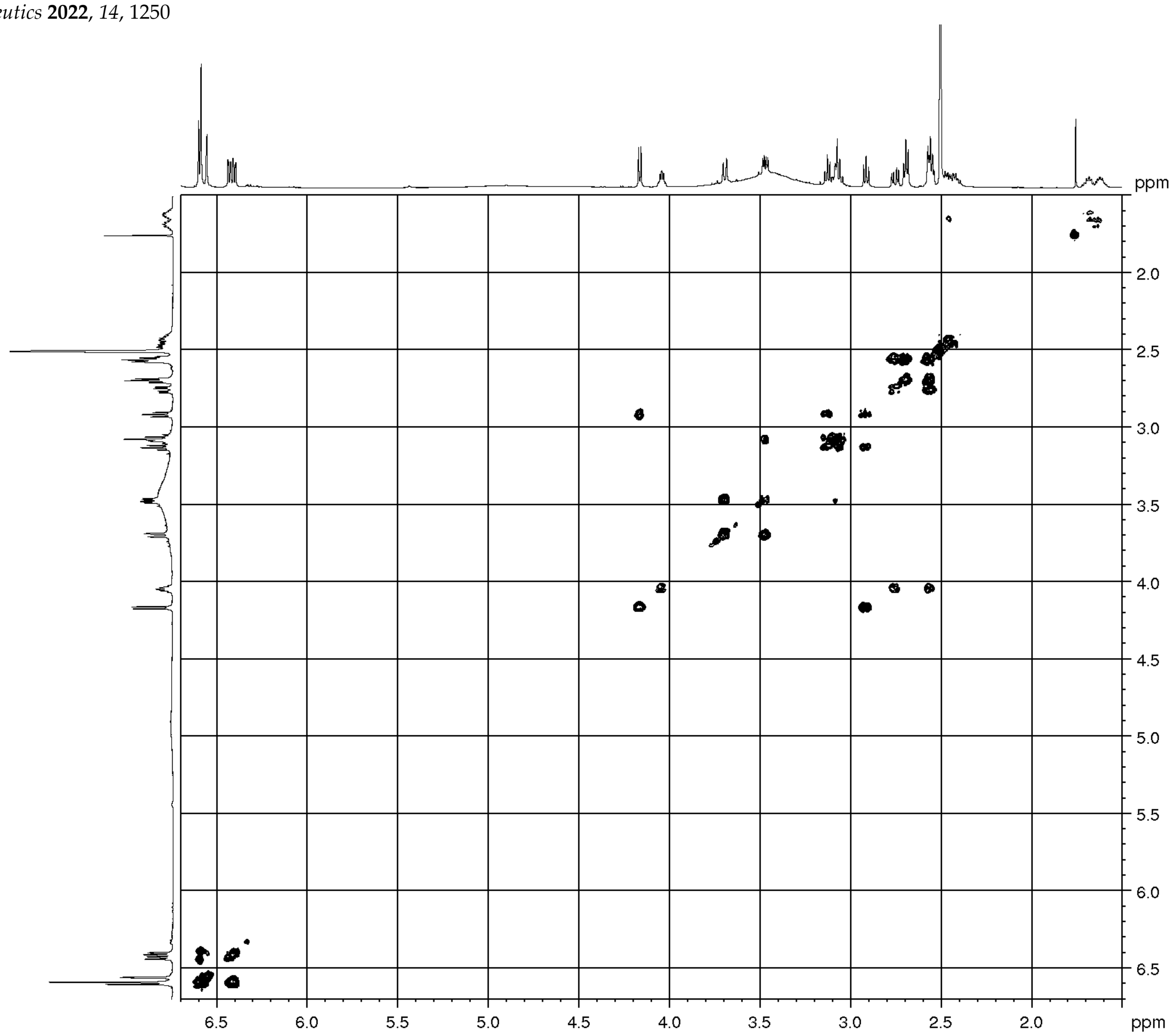

Figure S3. COSY spectrum of hirsutanolol-5-*O*-β-D-glucopyranoside (**1**) (DMSO-*d*6).

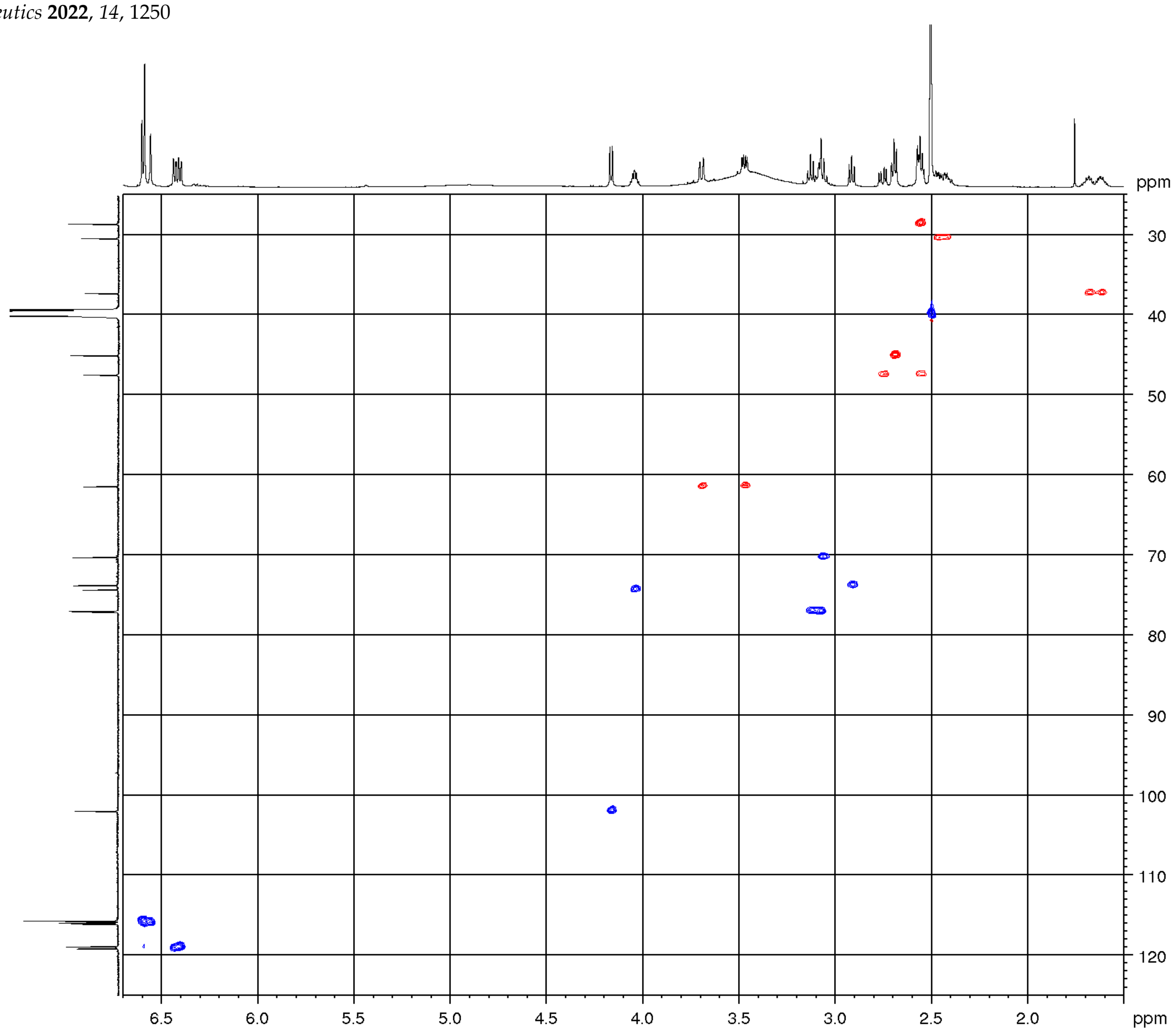

Figure S4. HSQC spectrum of hirsutanolol-5-*O*-β-D-glucopyranoside (**1**) (DMSO-d_6, 295 K).

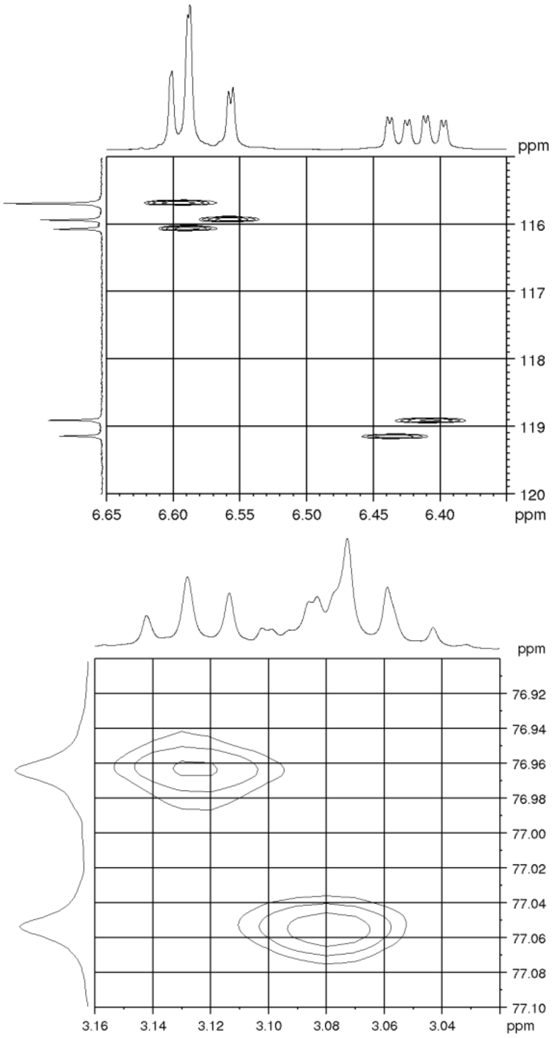

Figure S5. Band Selective HSQC spectra of hirsutanolol-5-*O*-β-D-glucopyranoside (**1**) (DMSO-d_6, 295 K).

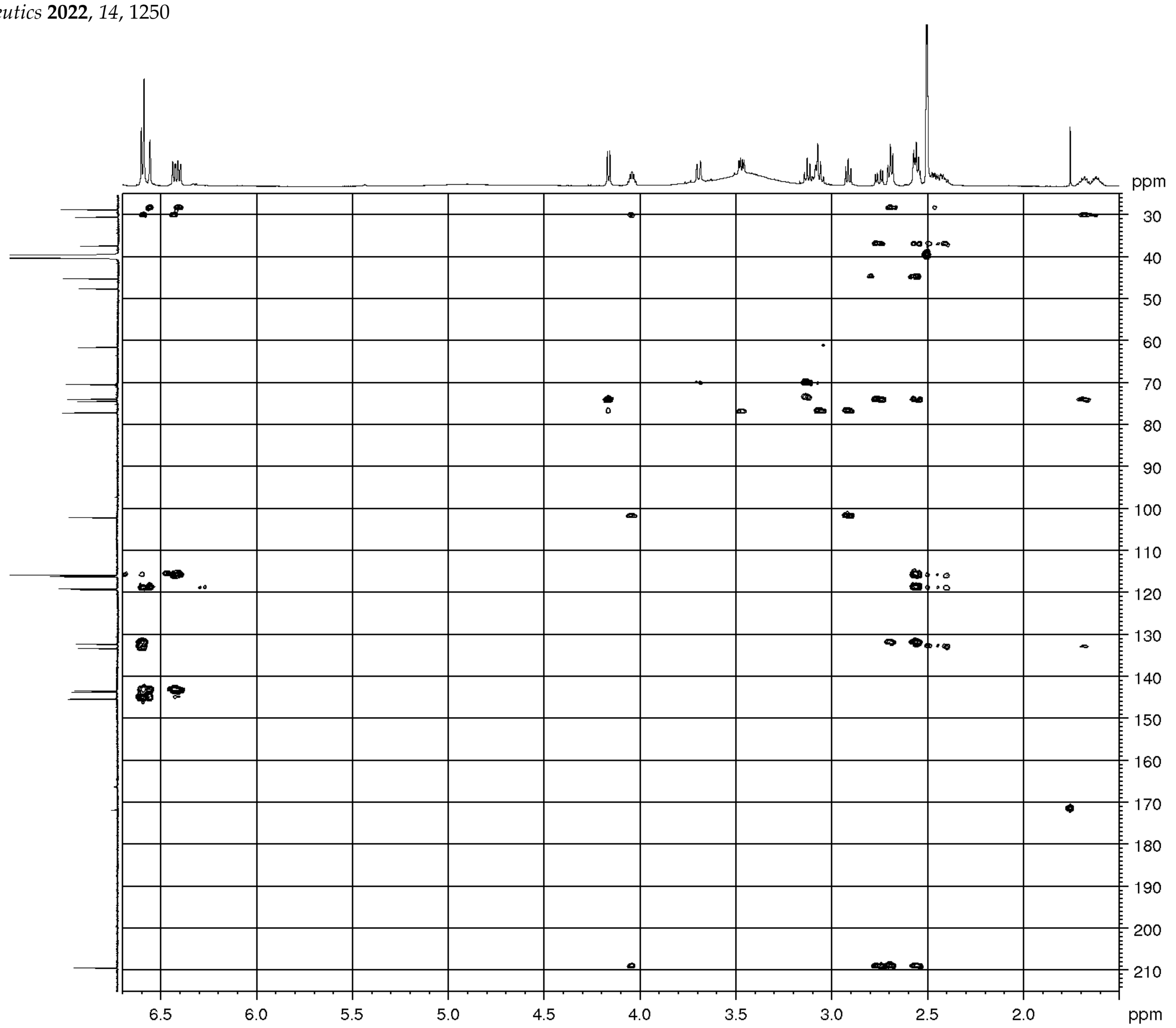

Figure S6. HMBC spectrum of hirsutanolol-5-*O*-β-D-glucopyranoside (**1**) (DMSO-d_6, 295 K).

NMR data of compound **4** (platyphyllonol-5-*O*-β-D- xylopyranoside): ^{1}H NMR (DMSO-d_6, 600 MHz, 295 K): δ 9.14 (m, 2H, OH-4′, OH-4″), 6.97 (~d, $^{3}J_{H,H}$=8.5 Hz, 2H, H-2′, H-6′), 6.94 (~d, $^{3}J_{H,H}$=8.5 Hz, 2H, H-2″, H-6″), 6.64 (m, 4H, H-3′, H-5′, H-3″, H-5″), 4.94 (m, 3H, OH_{Xyl}-2, OH_{Xyl}-3, OH_{Xyl}-4), 4.12 (d, $^{3}J_{H,H}$=7.7 Hz, 1H, H_{Xyl}-1), 3.99 (m, 1H, H-5), 3.68 (dd, $^{2}J_{H,H}$=11.4 Hz, $^{3}J_{H,H}$=5.4 Hz, 1H, H_{Xyl}-6a), 3.27 (m, 1H, H_{Xyl}-4), 3.07 (t, $^{3}J_{H,H}$=8.9 Hz, 1H, H_{Xyl}-3), 3.01 (m, 1H, H_{Xyl}-6b), 2.90 (m, 1H, H_{Xyl}-2), 2.75 (dd, $^{2}J_{H,H}$=16.3 Hz, $^{3}J_{H,H}$=6.3 Hz, 1H, H-4a), 2.72 (m, 2H, H-2), 2.63 (m, 2H, H-1), 2.56 (m, 1H, H-4b), 2.52 (m, 1H, H-7a), 2.43 (m, 1H, H-7b), 1.63 (m, 2H, H-6). ^{13}C NMR (DMSO-d_6, 150 MHz, 295 K): δ 209.1 (C-3), 155.6 (C-4′), 155.4 (C-4″), 132.2 (C-1″), 131.3 (C-1′), 129.3 (C-2′, C-6′ C-2″, C-6″), 115.2 (C-3′, C-5′, C-3″, C-5″), 102.7 (C_{Xyl}-1), 76.9 (C_{Xyl}-3), 74.3 (C-5), 73.5 (C_{Xyl}-2), 69.8 (C_{Xyl}-4), 66.0 (C_{Xyl}-5), 47.4 (C-4), 45.0 (C-2), 37.4 (C-6), 30.2 (C-7), 28.3 (C-1).

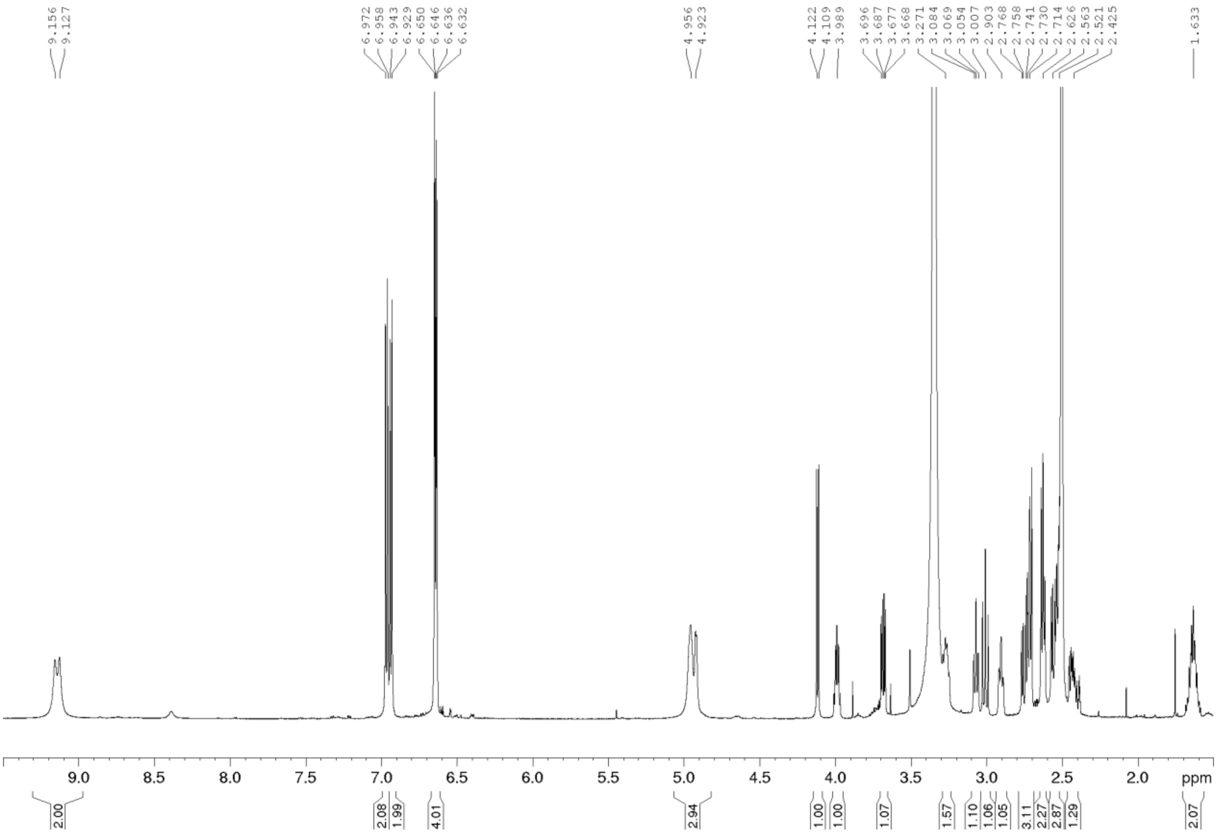

Figure S7. ^{1}H NMR spectrum of platyphyllonol-5-*O*-β-D- xylopyranoside (**4**) (DMSO-*d*6, 295 K).

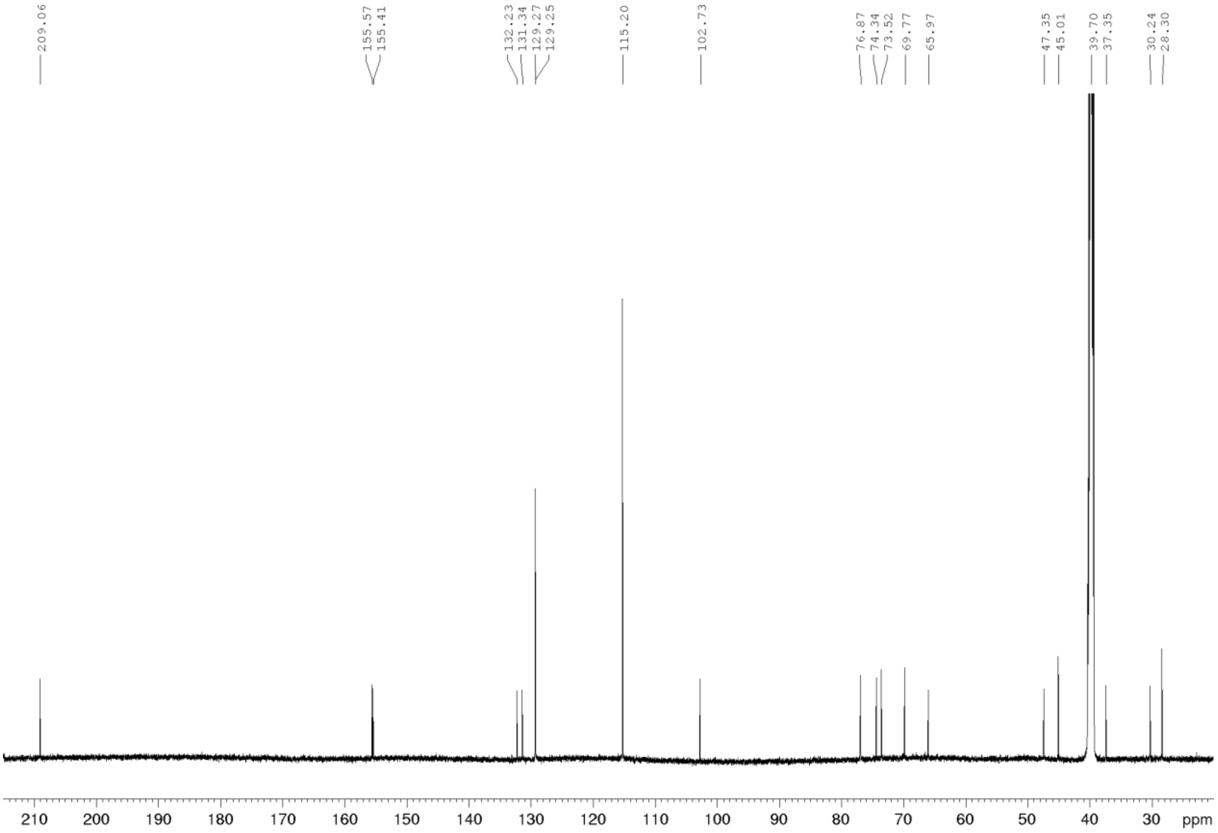

Figure S8. ^{13}C NMR spectrum of platyphyllonol-5-*O*-β-D- xylopyranoside (**4**) (DMSO-*d*6, 295 K).

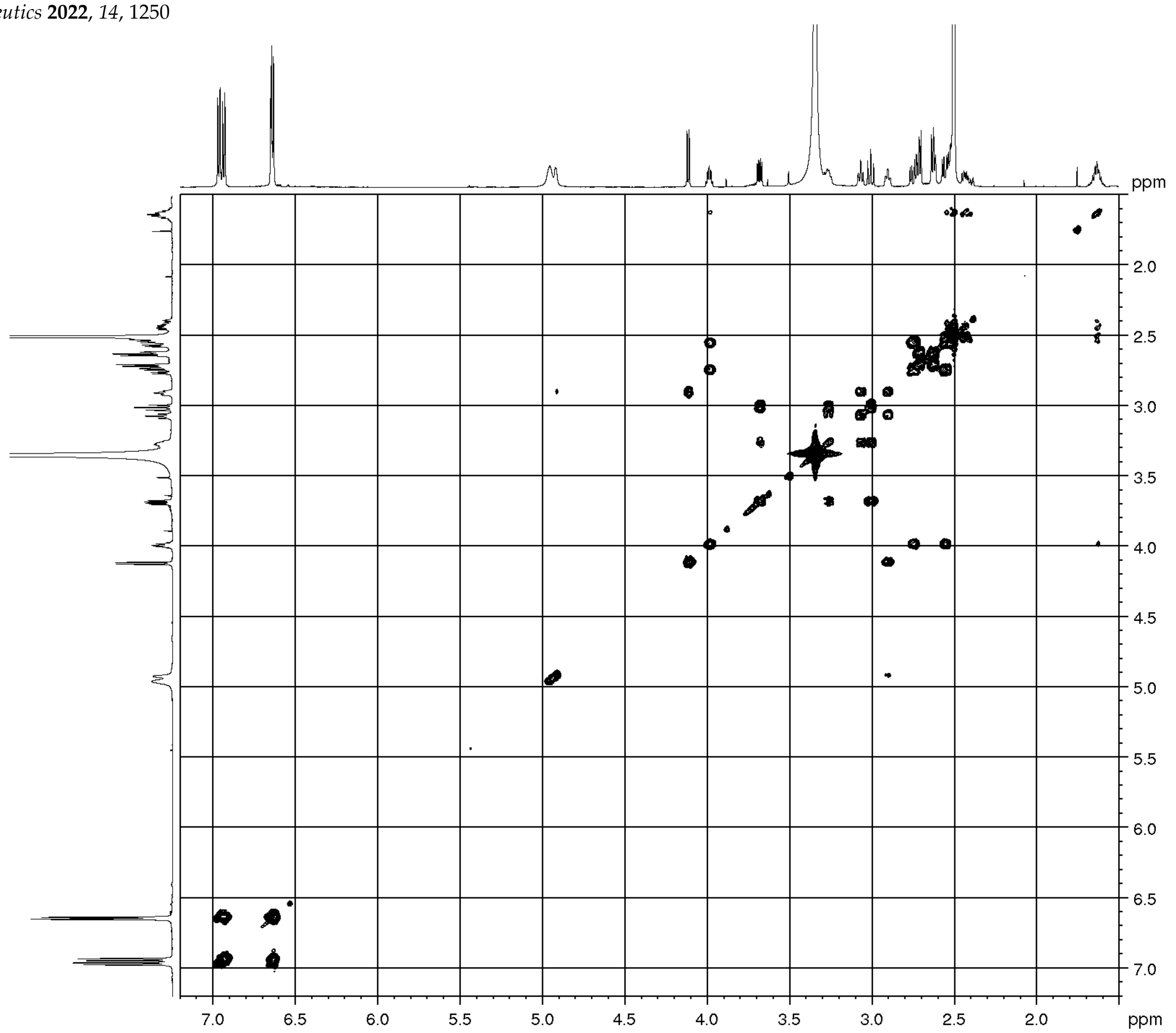

Figure S9. COSY spectrum of platyphyllonol-5-*O*-β-D- xylopyranoside (**4**) (DMSO-d_6, 295 K).

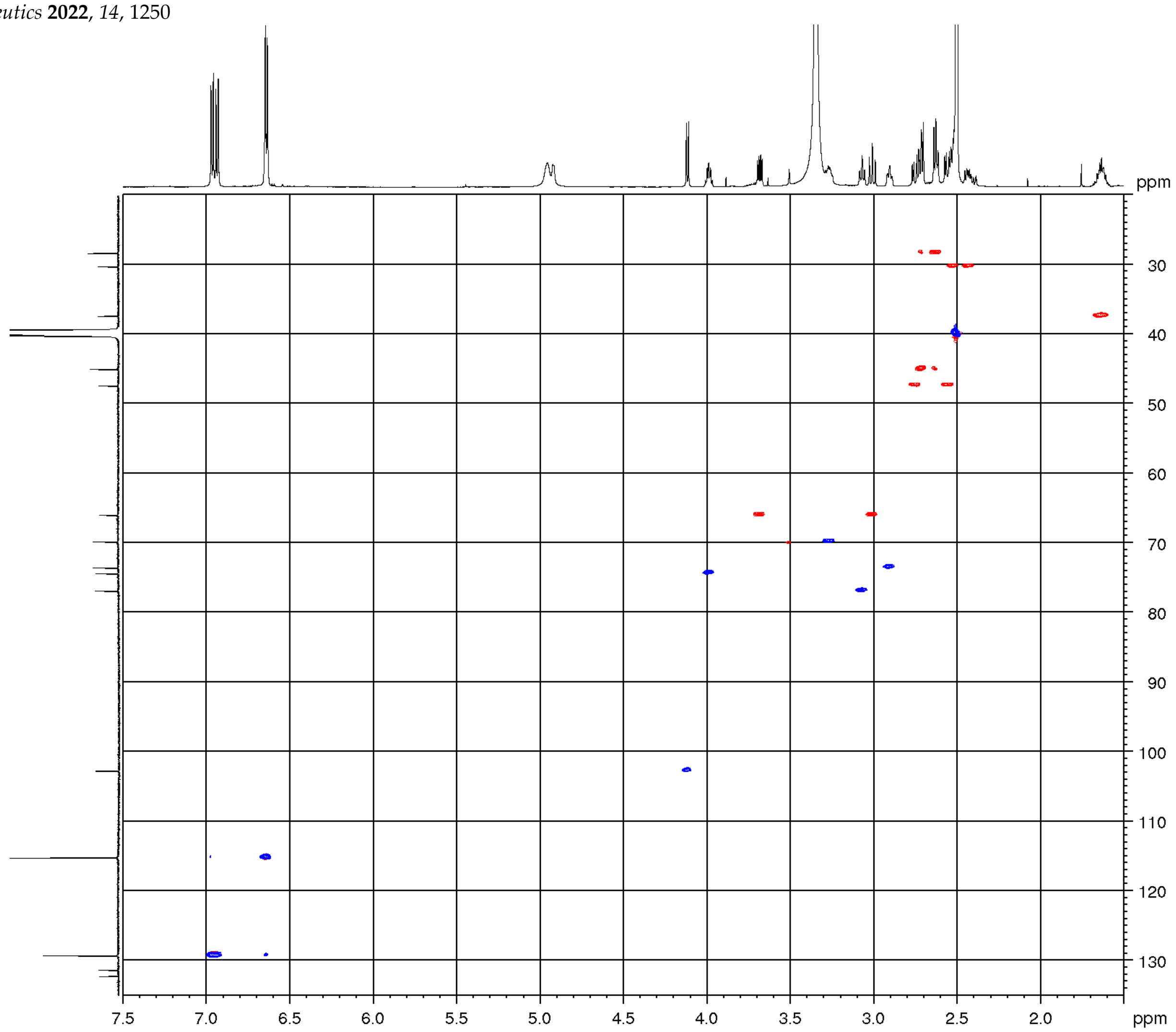

Figure S10. HSQC spectrum of platyphyllonol-5-*O*-β-D- xylopyranoside (**4**) (DMSO-d_6, 295 K).

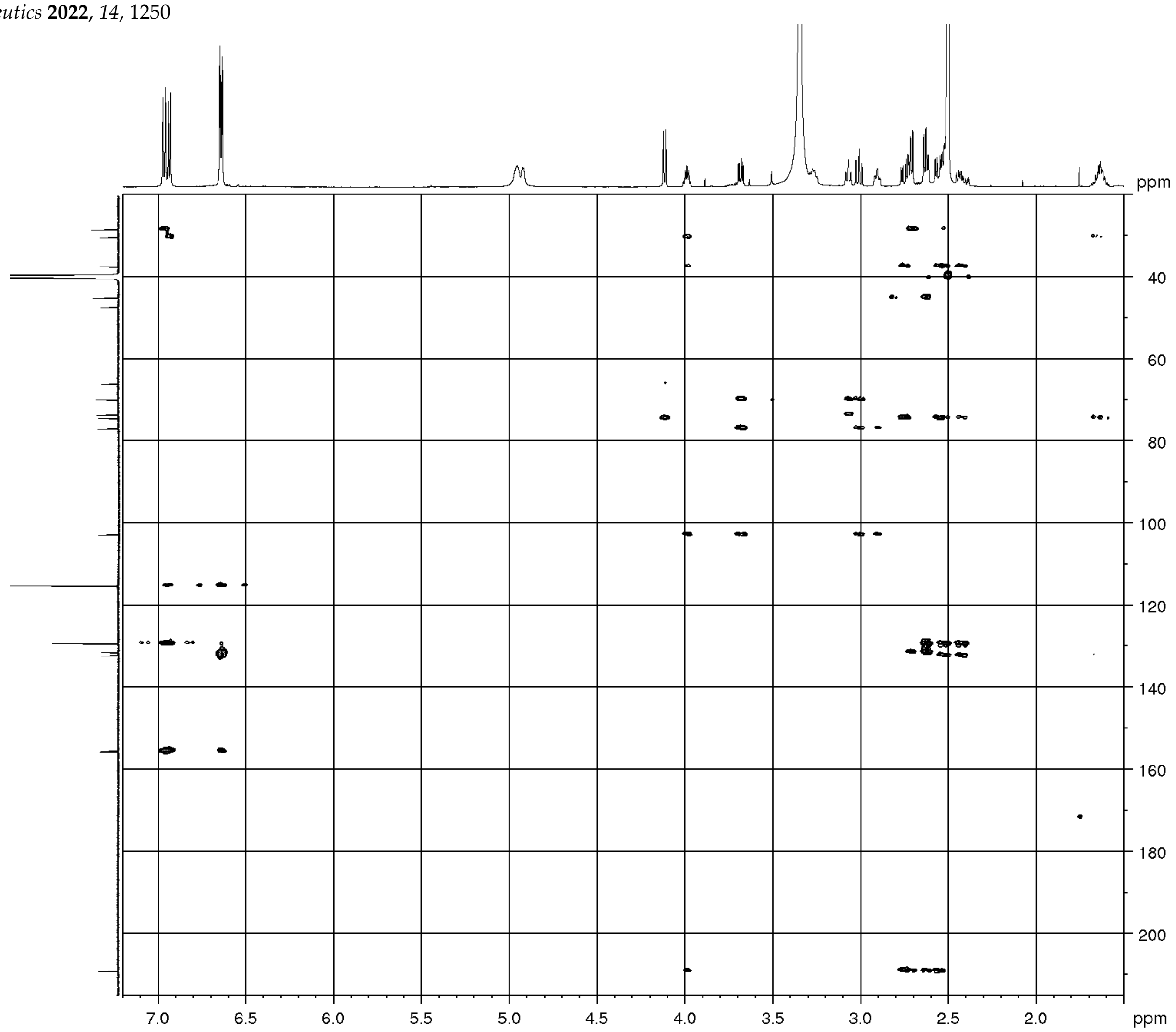

Figure S11. HMBC spectrum of platyphyllonol-5-*O*-β-D- xylopyranoside (**4**) (DMSO-d_6, 295 K).

NMR data of compound **5** (platyphyllenone): ^{1}H NMR (DMSO-d_6, 600 MHz, 295 K): δ 7.00 (~d, $^{3}J_{H,H}$=8.4 Hz, 2H, H-2′/H-2″, H-6′/H-6″), 6.98 (~d, $^{3}J_{H,H}$=8.4 Hz, H-2′/H-2″, H-6′/H-6″) 6.85 (dt, $^{2}J_{H,H}$=15.9 Hz, $^{3}J_{H,H}$=6.8 Hz, 1H, H-5), 6.67 (~d, $^{3}J_{H,H}$=8.4 Hz, 2H, H-3′/H-3″, H-5′/H-5″), 6.65 (~d, $^{3}J_{H,H}$=8.4 Hz, 2H H-3′/H-3″, H-5′/H-5″), 6.08 (dt, $^{2}J_{H,H}$=15.9 Hz, $^{4}J_{H,H}$=1.4 Hz, 1H, H-4), 2.79 (m, 2H, H-2), 2.67 (m, 2H, H-1), 2.62 (m, 2H, H-7), 2.43 (m, 2H, H-6). ^{13}C NMR (DMSO-d_6, 150 MHz, 295 K): δ 199.5 (C-3), 155.7 (C-4′, C-4″), 147.1 (C-5), 131.3 (C-1/C-1″), 131.1 (C-1/C-1″), 130.5 (C-4), 129.4 (C-6′/C-6″), 129.3 (C-6′/C-6″), 115.3 (C-5′/C-5″), 115.2 (C-5′/C-5″), 41.4 (C-2), 34.1 (C-6), 33.0 (C-7), 29.0 (C-1).

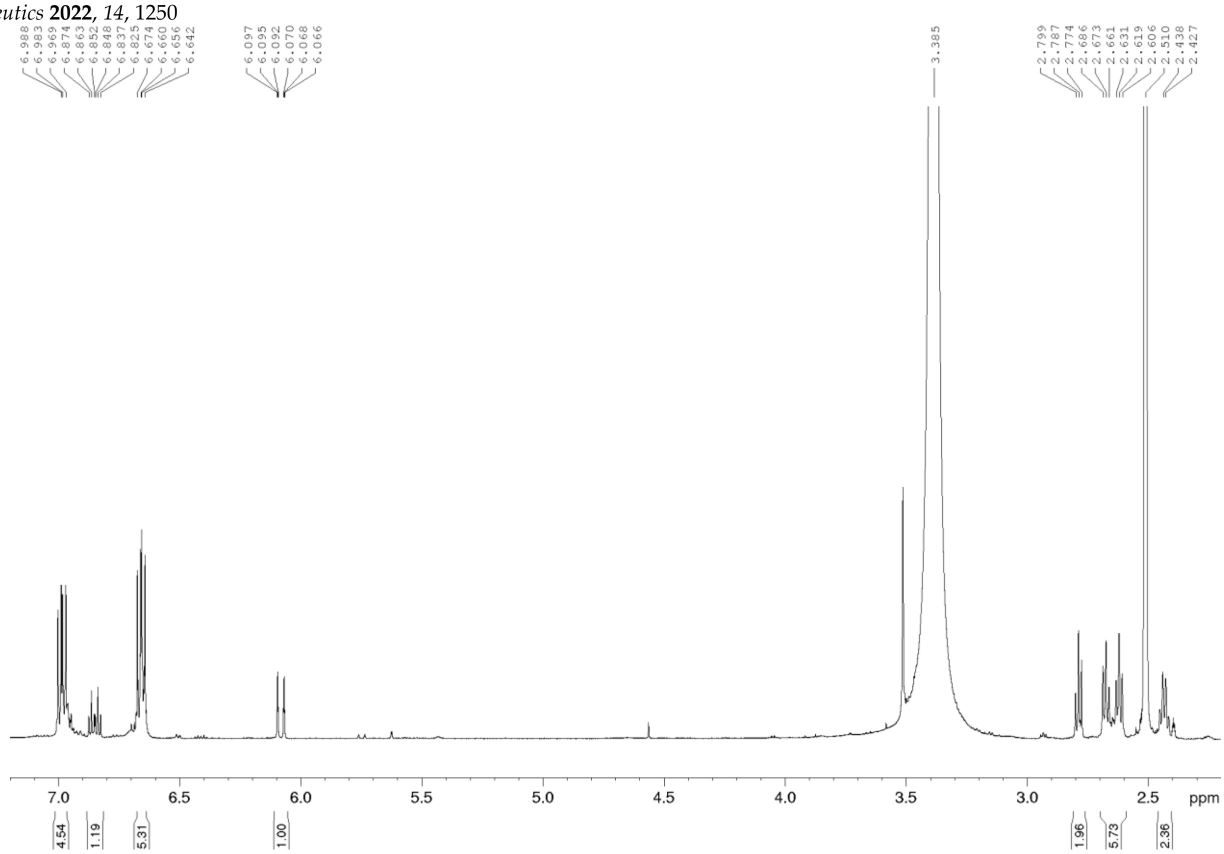

Figure S12. ^{1}H NMR spectrum of platyphyllenone (**5**) (DMSO-d_6, 295 K).

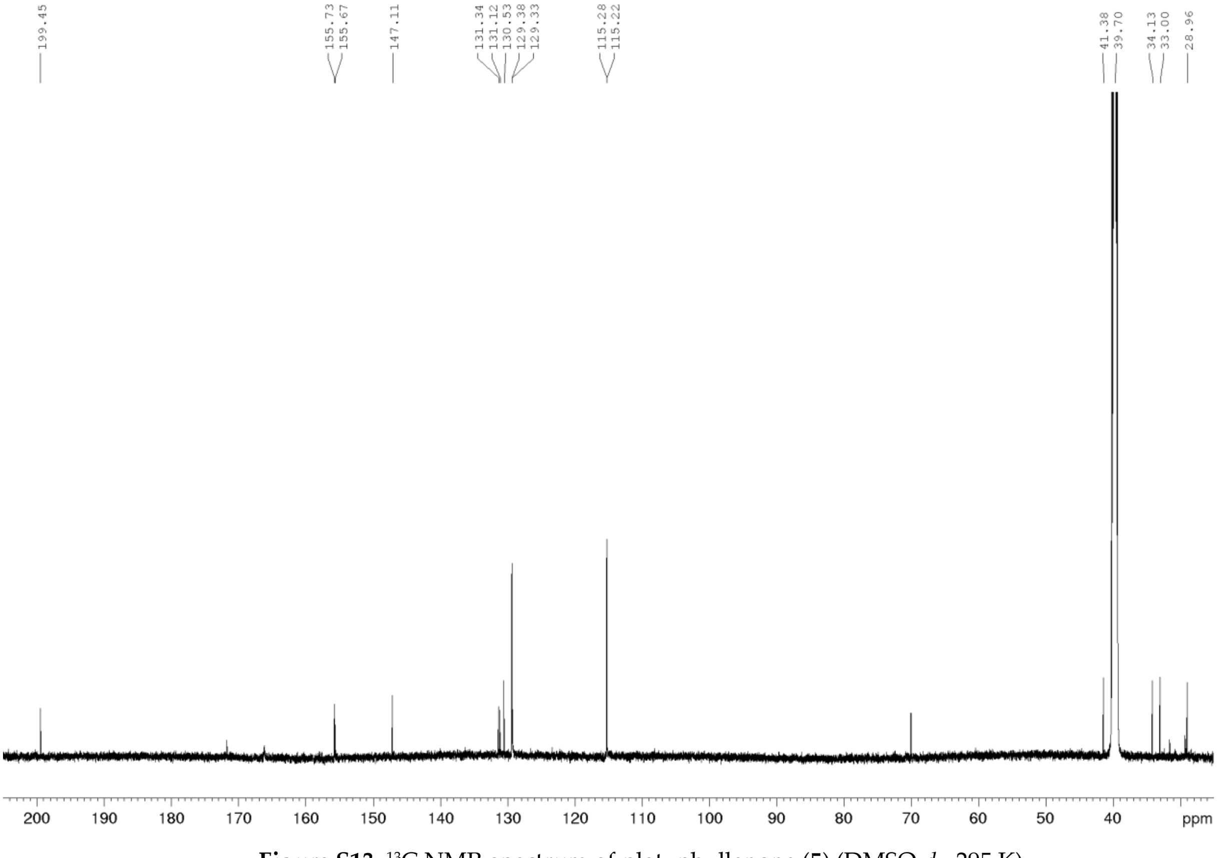

Figure S13. ^{13}C NMR spectrum of platyphyllenone (**5**) (DMSO-d_6, 295 K).

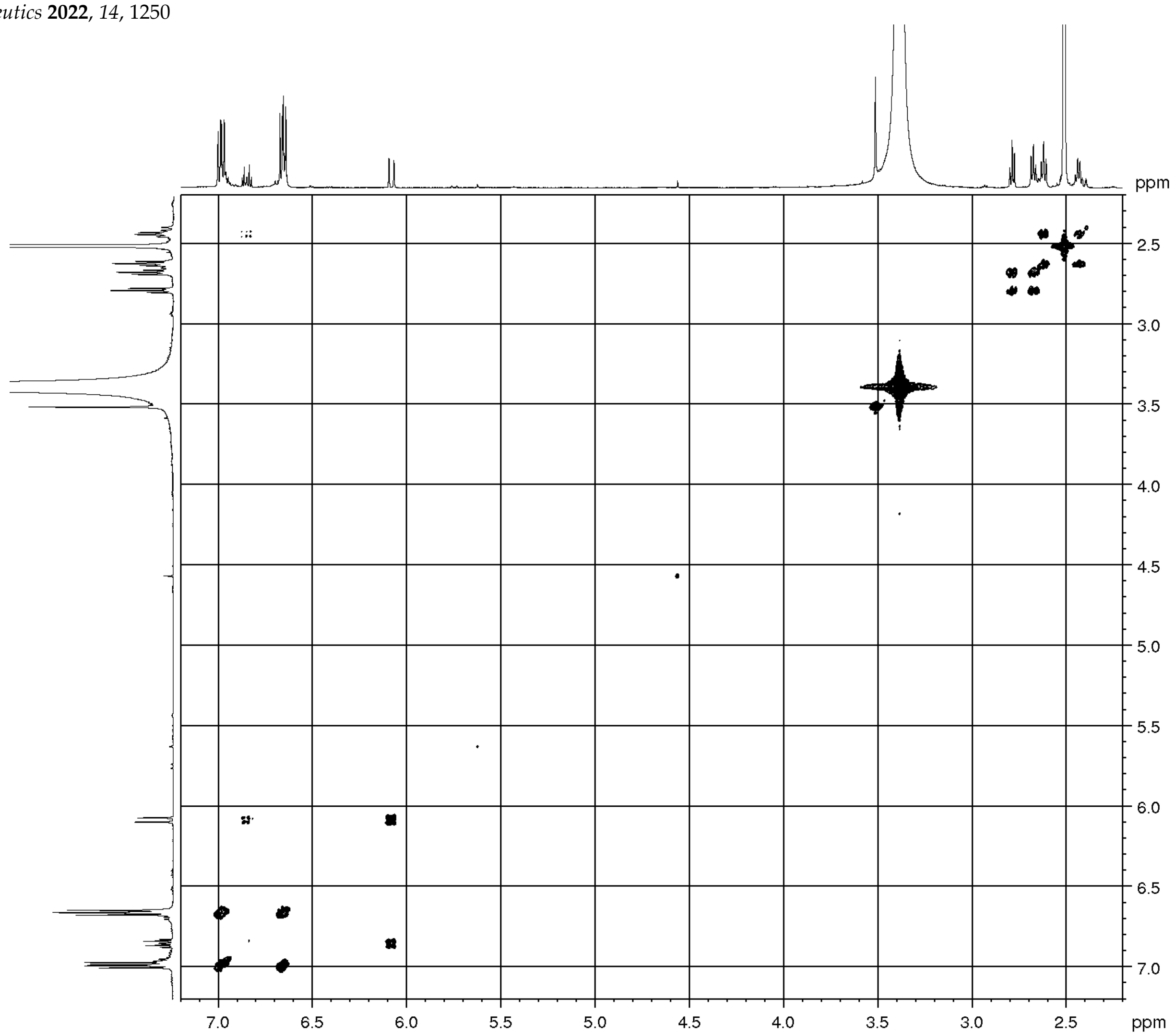

Figure S14. COSY spectrum of platyphyllenone (**5**) (DMSO-d_6, 295 K).

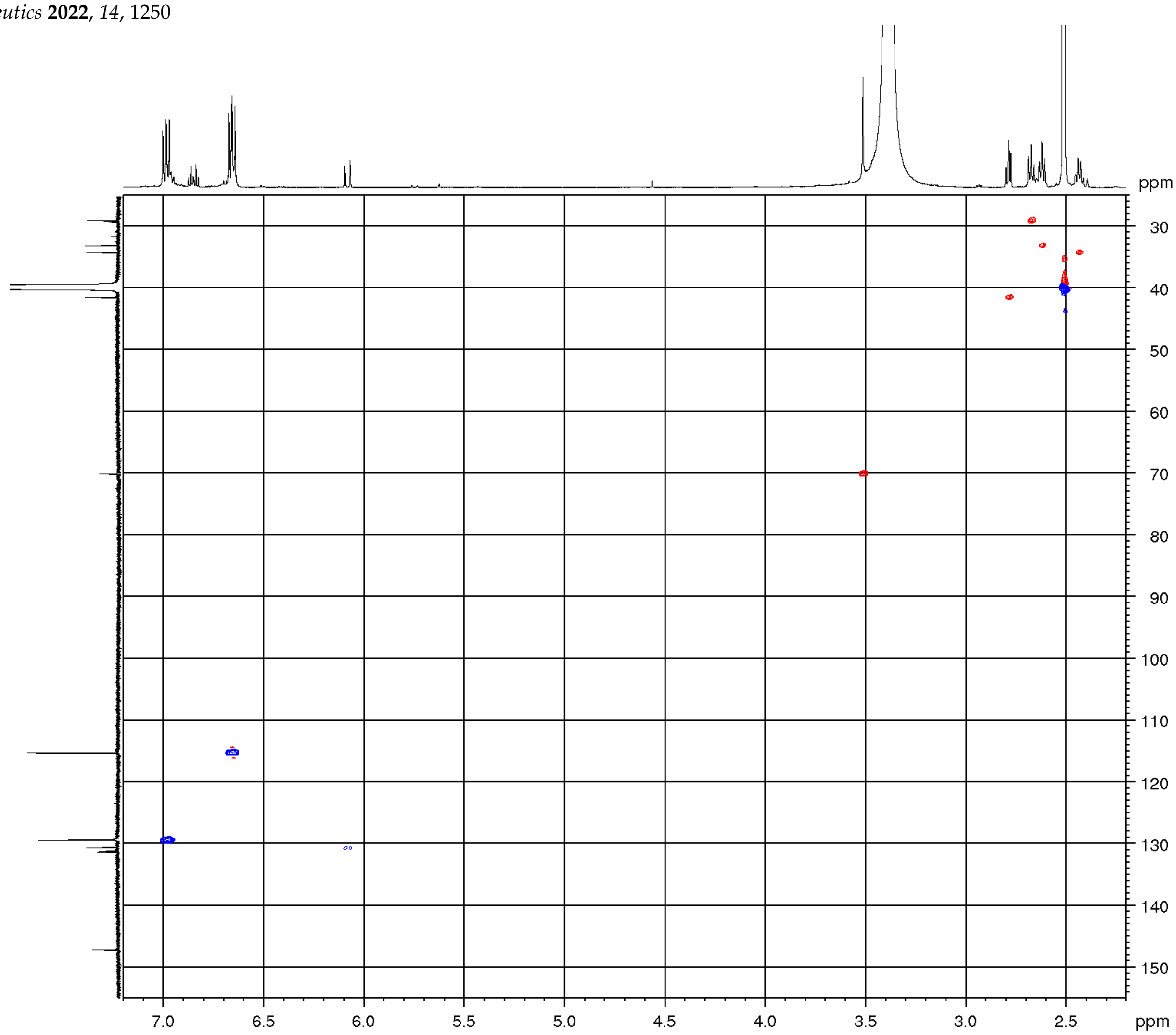

Figure S15. HSQC spectrum of platyphyllenone (**5**) (DMSO-d_6, 295 K).

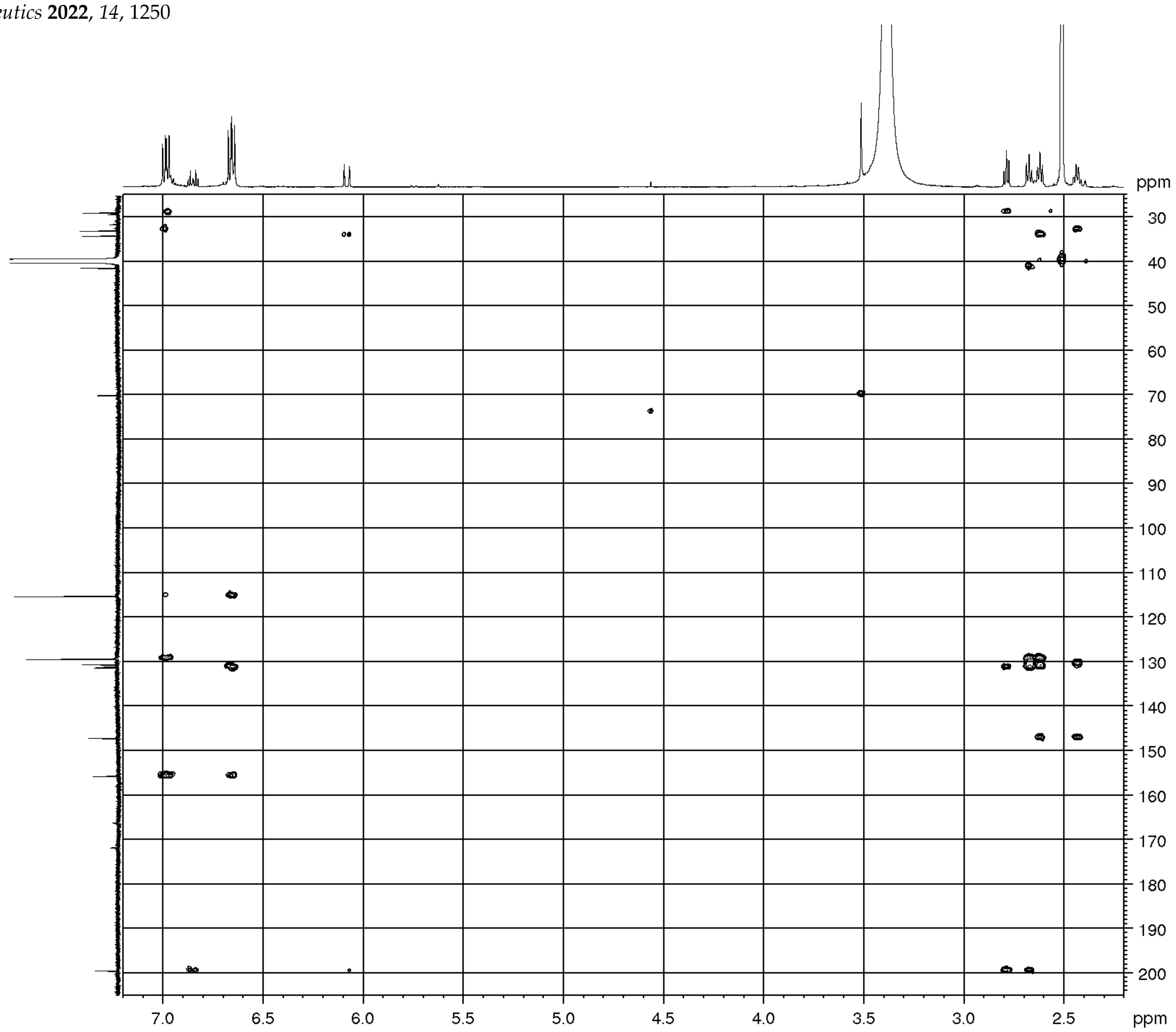

Figure S16. HMBC spectrum of platyphyllenone (**5**) (DMSO-d_6, 295 K).

NMR data of compound **6** (alnusonol-11-*O*-β-D-glucopyranoside): ^{1}H NMR (DMSO-d_6, 600 MHz, 295 K): δ 6.98 (m, 1H, H-15), 6.96 (m, 1H, H-5), 6.71 (d, $^3J_{H,H}$=8.2 Hz, 1H, H-4), 6.70 (d, $^3J_{H,H}$=8.2 Hz, 1H, H-16), 6.62 (d, $^4J_{H,H}$=2.0 Hz, 1H, H-18), 6.49 (d, $^4J_{H,H}$=2.0 Hz, 1H, H-19), 4.24 (d, $^3J_{H,H}$=7.8 Hz, 1H, H_{Glc}-1), 4.08 (m, 1H, H-11), 3.49 (dd, $^2J_{H,H}$=11.6 Hz, $^3J_{H,H}$=2.0 Hz, 1H, H_{Glc}-6a), 3.40 (dd, $^2J_{H,H}$=11.6 Hz, $^3J_{H,H}$=5.2 Hz, 1H, H_{Glc}-6b) 3.16 (m, 2H, H-10), 3.11 (m, 1H, H-8a), 3.10 (m, 1H, H_{Glc}-3), 3.09 (m, 1H, H_{Glc}-4), 2.99 (m, 1H, H_{Glc}-5), 2.98 (m, 1H, H-13a), 2.91 (m, 1H, H-8b), 2.91 (m, 1H, H-6a), 2.88 (m, 1H, H_{Glc}-2), 2.81 (m, 1H, H-7b), 2.68 (m, 1H, H-13b), 2.22 (m, 1H, H-12a), 1.89 (m, 1H, H-12b). ^{13}C NMR (DMSO-d_6, 150 MHz, 295 K): δ 210.5 (C-9), 153.2 (C-3), 153.0 (C-17), 133.2 (C-19), 132.4 (C-18), 130.5 (C-14), 129.5 (C-6), 129.3 (C-15), 128.2 (C-5), 126.4 (C-1, C-2), 116.4 (C-16), 116.3 (C-4), 102.2 (C_{Glc}-1), 76.9 (C_{Glc}-4), 76.4 (C_{Glc}-5), 74.8 (C-11), 73.7 (C_{Glc}-2), 70.1 (C_{Glc}-3), 61.2 (C_{Glc}-6), 51.5 (C-10), 41.6 (C-8), 33.8 (C-12), 28.0 (C-13), 26.4 (C-7).

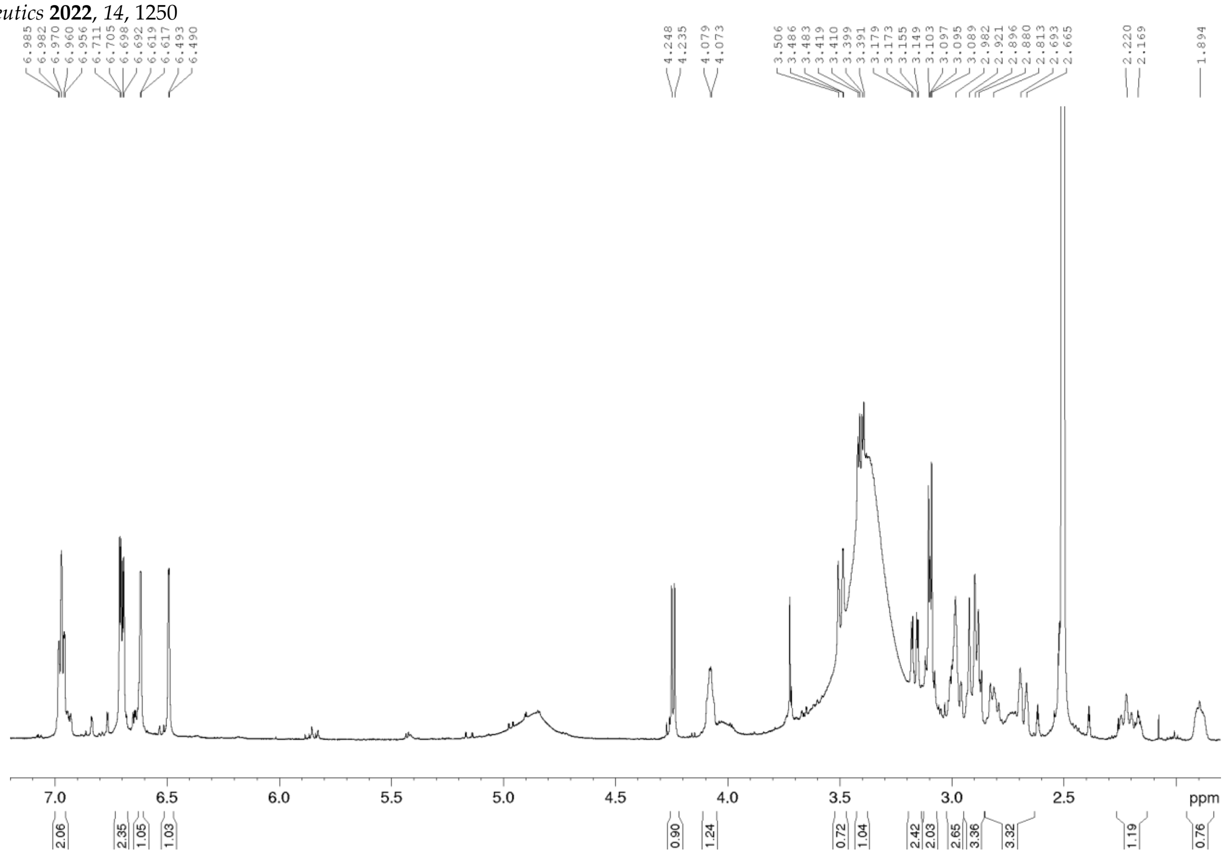

Figure S17. ^{1}H NMR spectrum of alnusonol-11-*O*-β-D-glucopyranoside (**6**) (DMSO-d_6, 295 K).

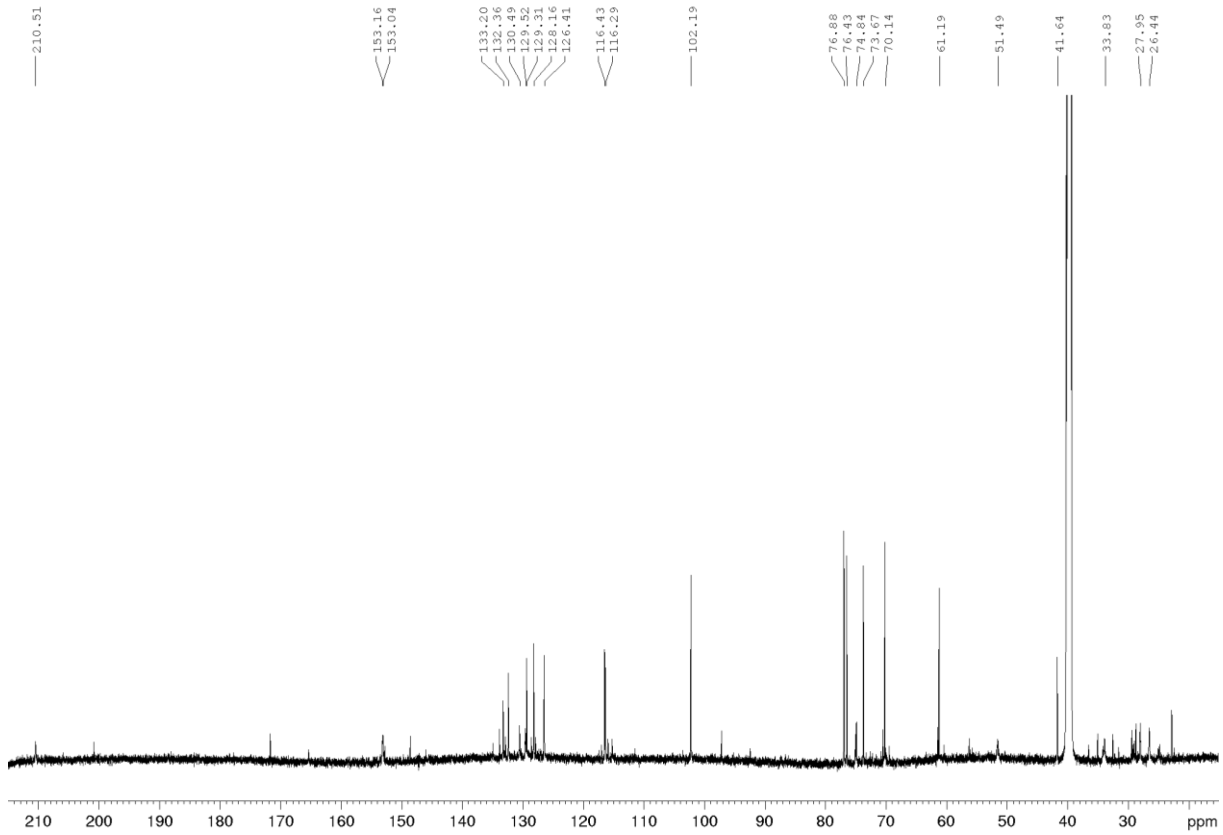

Figure S18. ^{13}C NMR spectrum of alnusonol-11-*O*-β-D-glucopyranoside (**6**) (DMSO-d_6, 295 K).

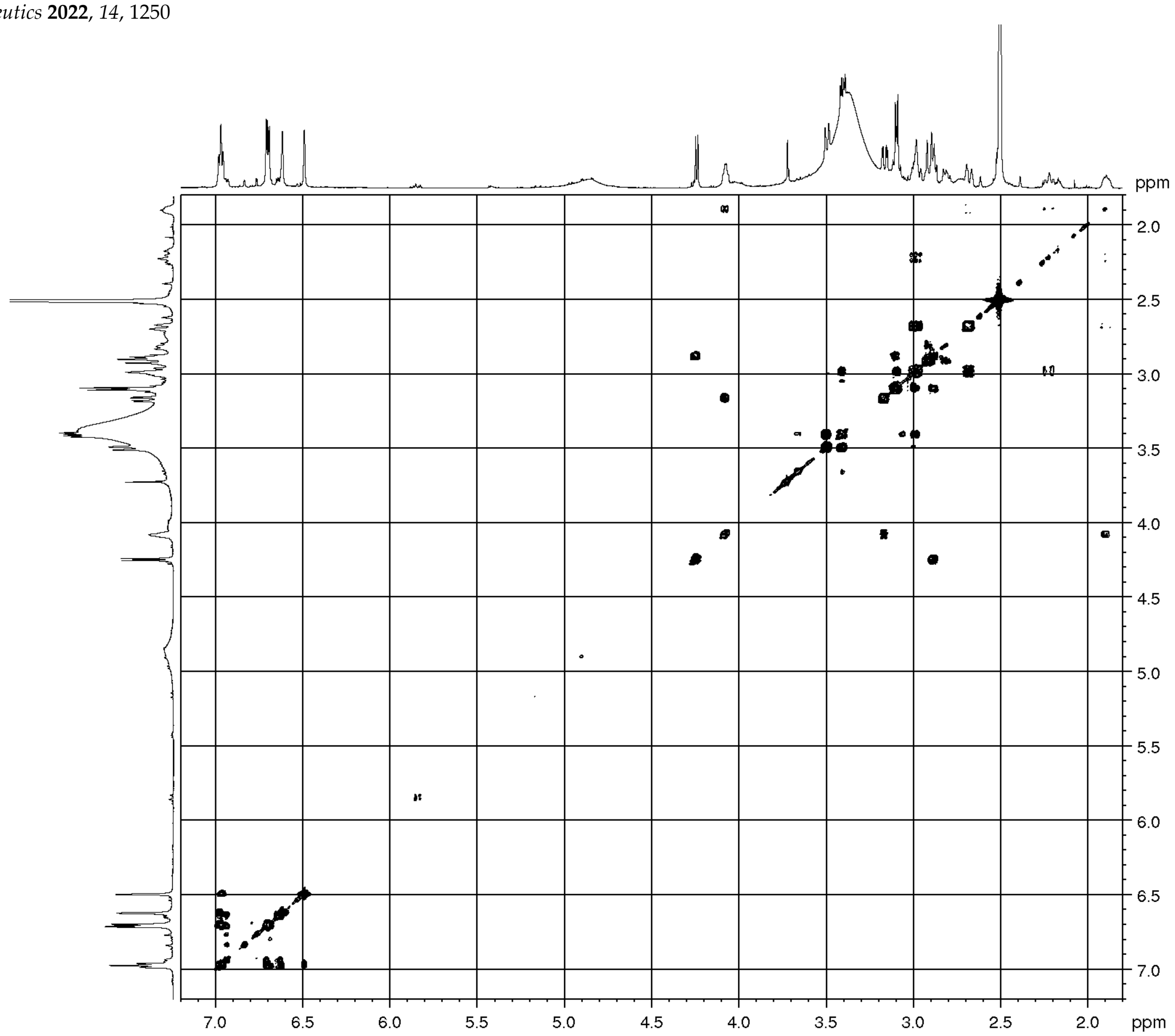

Figure S19. COSY spectrum of alnusonol-11-*O*-β-D-glucopyranoside (**6**) (DMSO-d_6, 295 K).

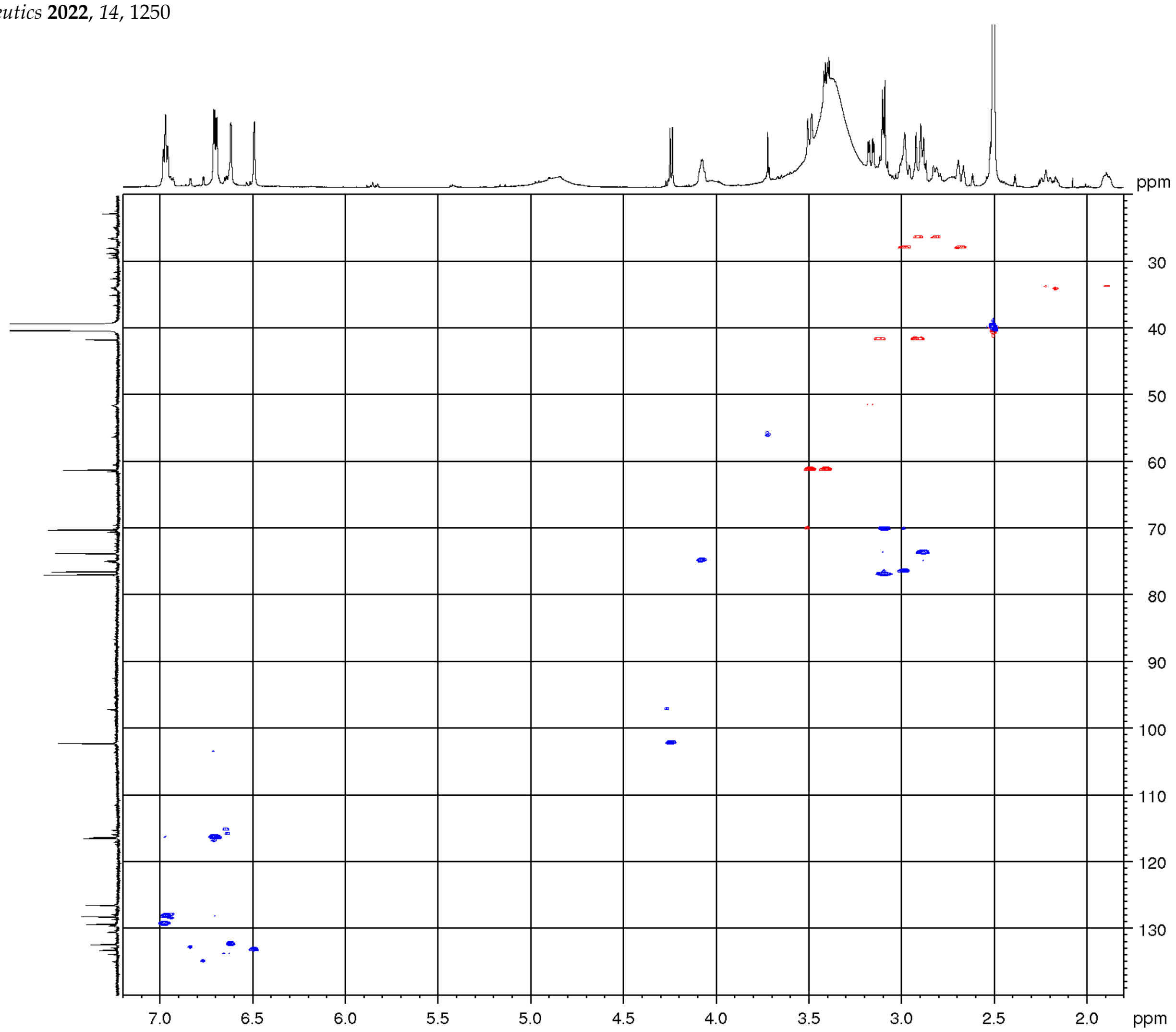

Figure S20. HSQC spectrum of alnusonol-11-*O*-β-D-glucopyranoside (**6**) (DMSO-d_6, 295 K).

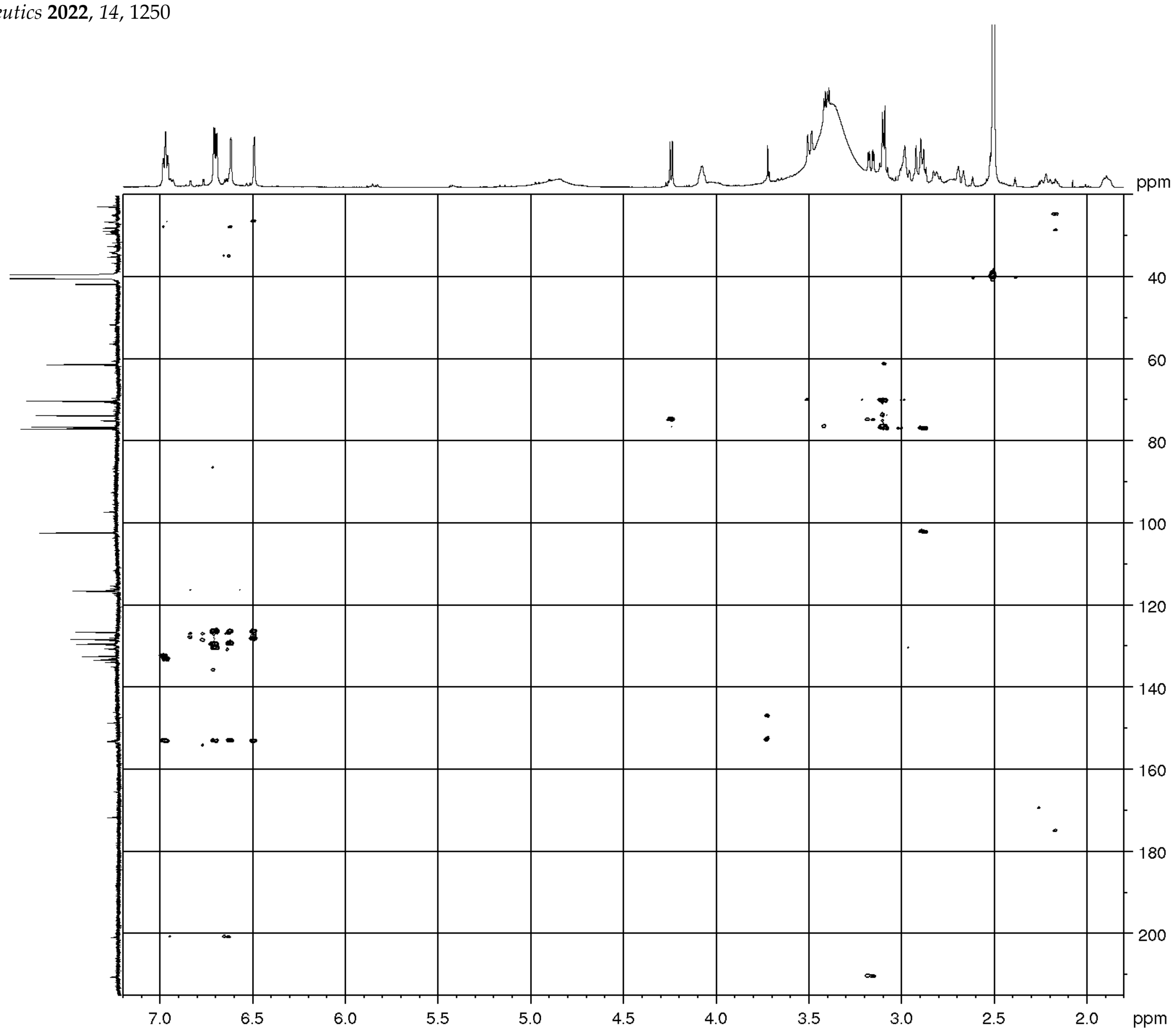

Figure S21. HMBC spectrum alnusonol-11-*O*-β-D-glucopyranoside (**6**) (DMSO-d_6, 295 K).

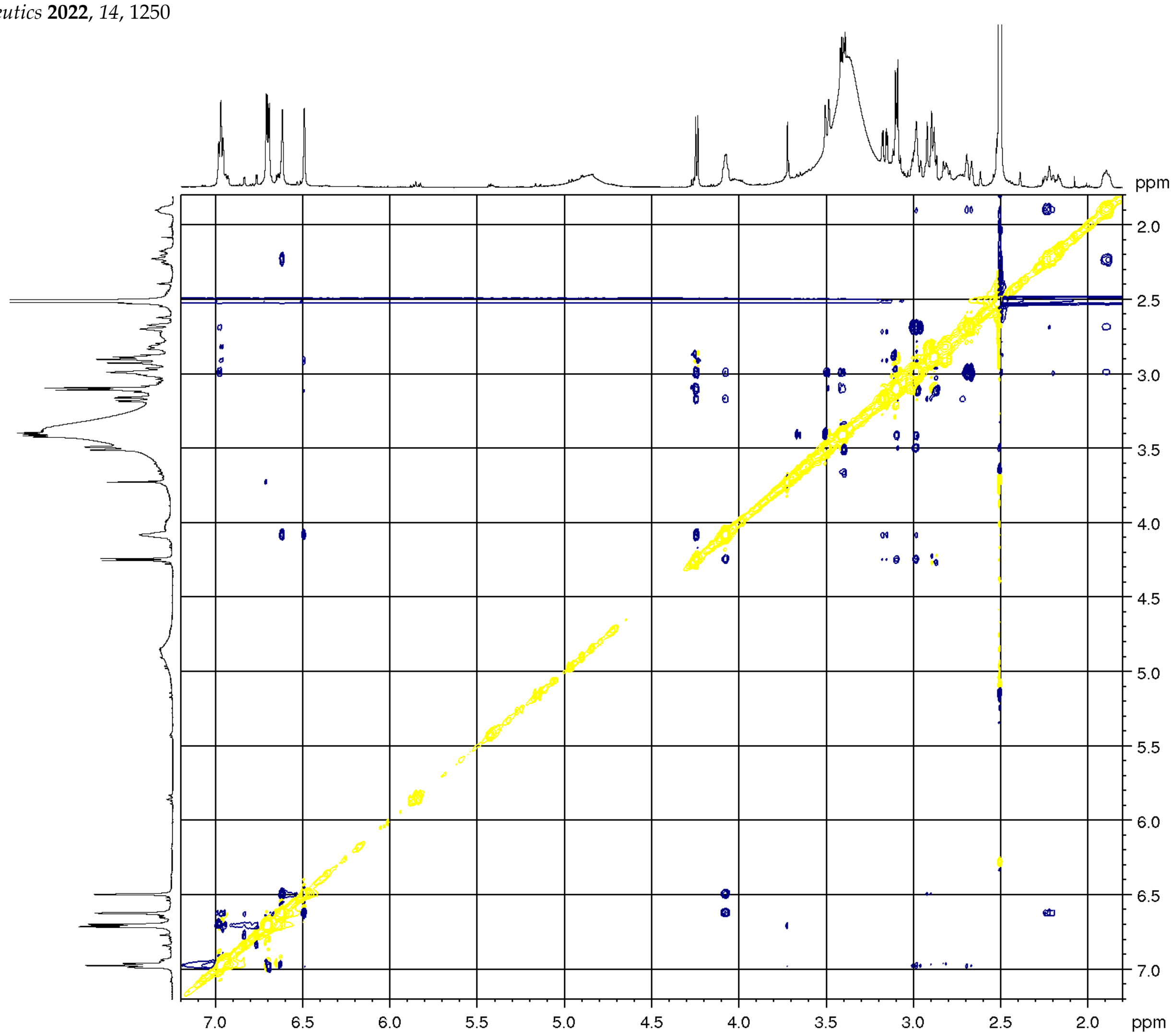

Figure S22. ROESY spectrum of alnusonol-11-*O*-β-D-glucopyranoside (**6**) (DMSO-*d*$_6$, 295 K).

NMR data of compound **7** (alnusone): ^{1}H NMR (DMSO-*d*$_6$, 600 MHz, 333 K): δ 6.95 (dt, $^2J_{H,H}$=15.5 Hz, $^3J_{H,H}$=7.5 Hz, H-11), 6.82 (m, 1H, H-19), 6.81 (m, 1H, H-18), 6.78 (m, 1H, H-15), 6.77 (m, 1H, H-5), 6.60 (dt, $^2J_{H,H}$=15.5 Hz, $^4J_{H,H}$=1.2 Hz, H-10), 6.49 (d, $^3J_{H,H}$=7.9 Hz, 1H, H-4), 6.42 (d, $^3J_{H,H}$=7.9 Hz, 1H, H-16), 2.98 (m, 2H, H-7), 2.80 (m, 2H, H-13), 2.70 (m, 2H, H-8), 2.53 (m, 2H, H-12). ^{13}C NMR (DMSO-*d*$_6$, 150 MHz, 333 K): δ 200.6 (C-9), 148.3 (C-11), 133.5 (C-10), 133.1 (C-18), 131.7 (C-19), 127.4 (C-15), 126.7 (C-5), 117.6 (C-4), 116.1 (C-16), 39.8 (C-8), 34.9 (C-12), 32.5 (C-13), 29.5 (C-7). (The C-1, C-2, C-3, C-6, C-14 and C-17 signals were broad even on 333 K.)

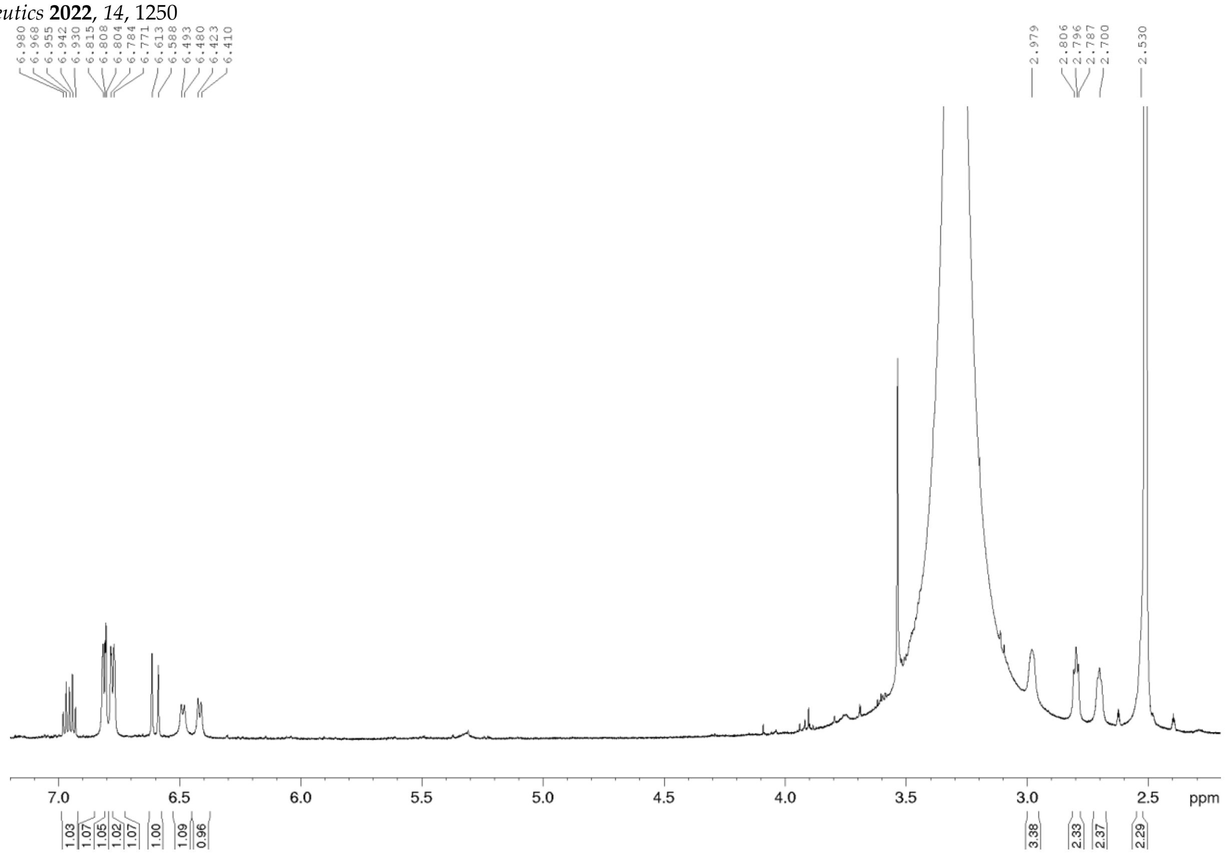

Figure S23. ^{1}H NMR spectrum of alnusone (**7**) (DMSO-d_6, 333 K).

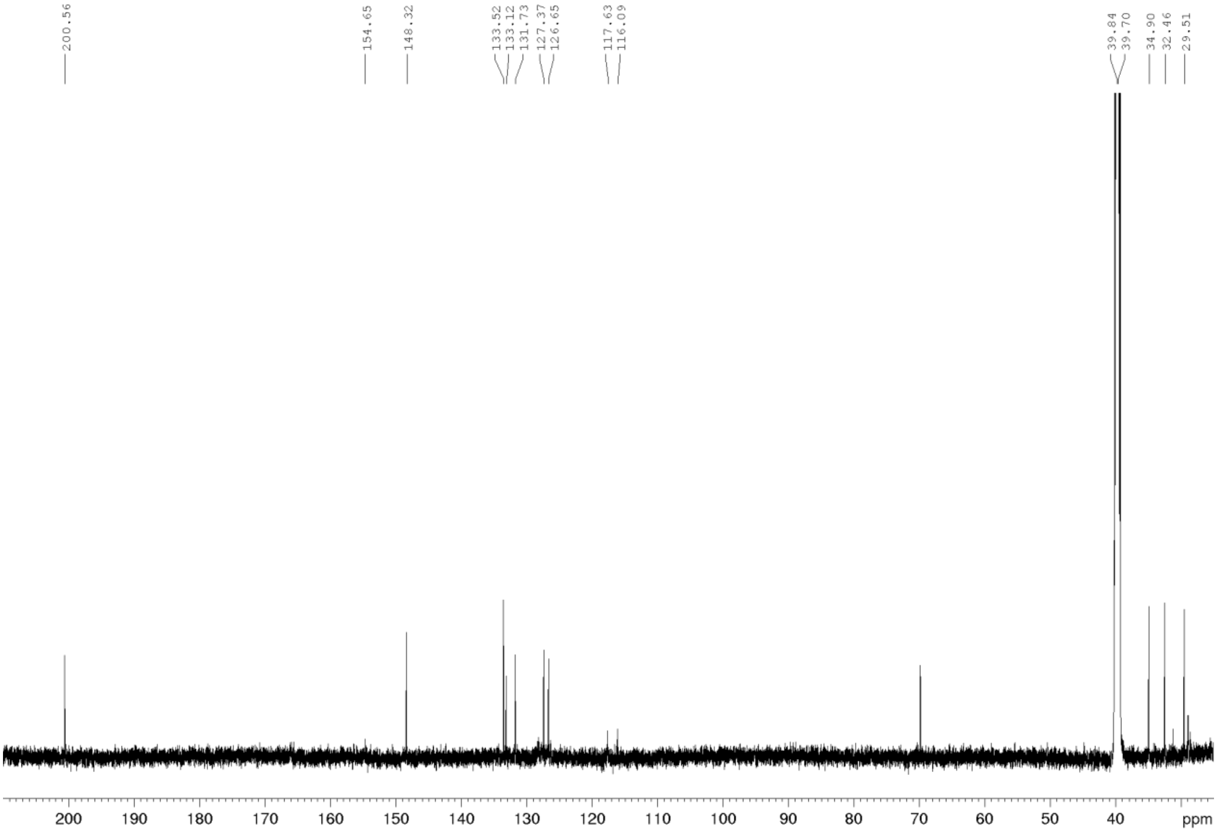

Figure S24. ^{13}C NMR spectrum of alnusone (**7**) (DMSO-d_6, 333 K).

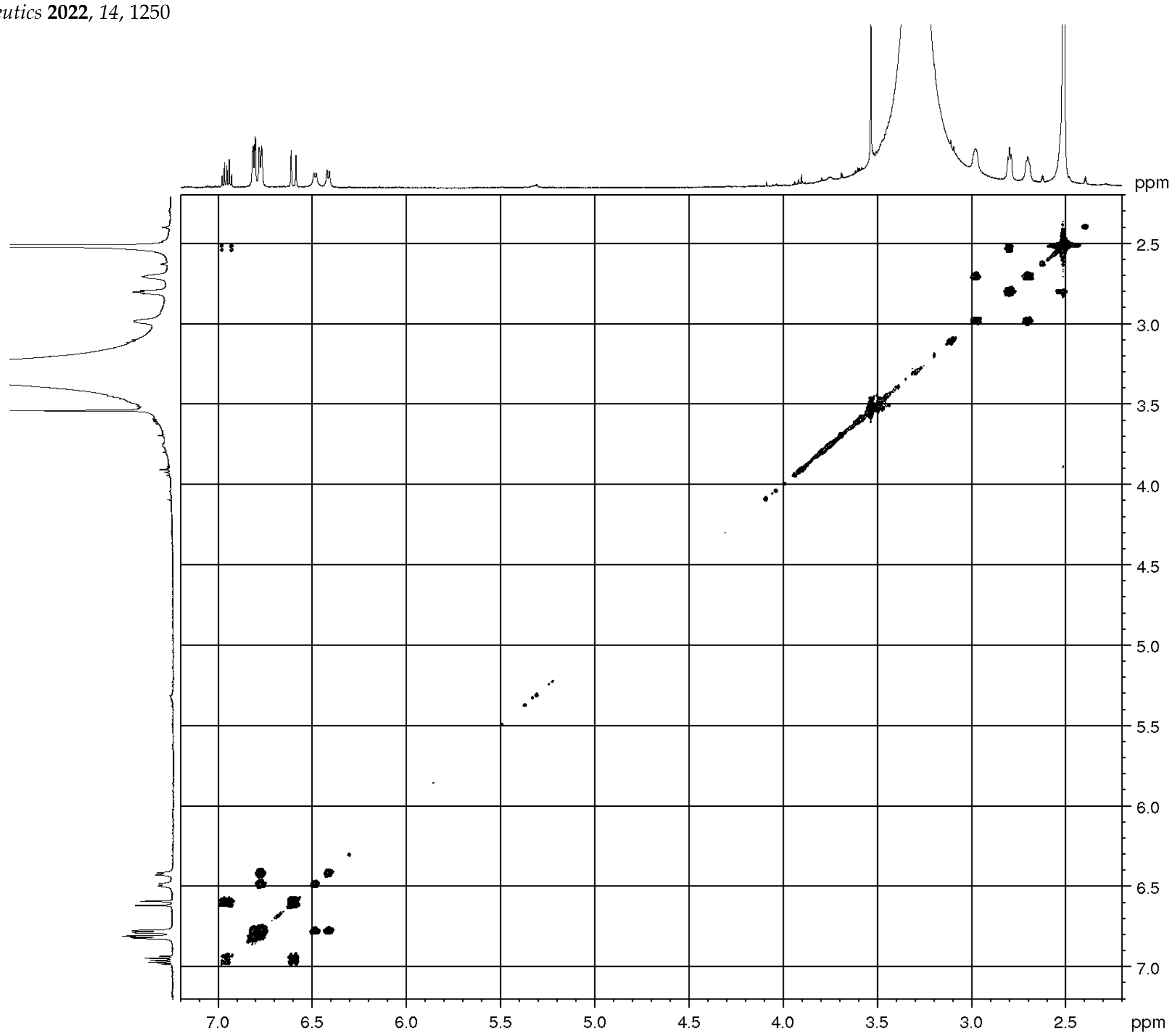

Figure S25. COSY spectrum of alnusone (**7**) (DMSO-d_6, 333 K).

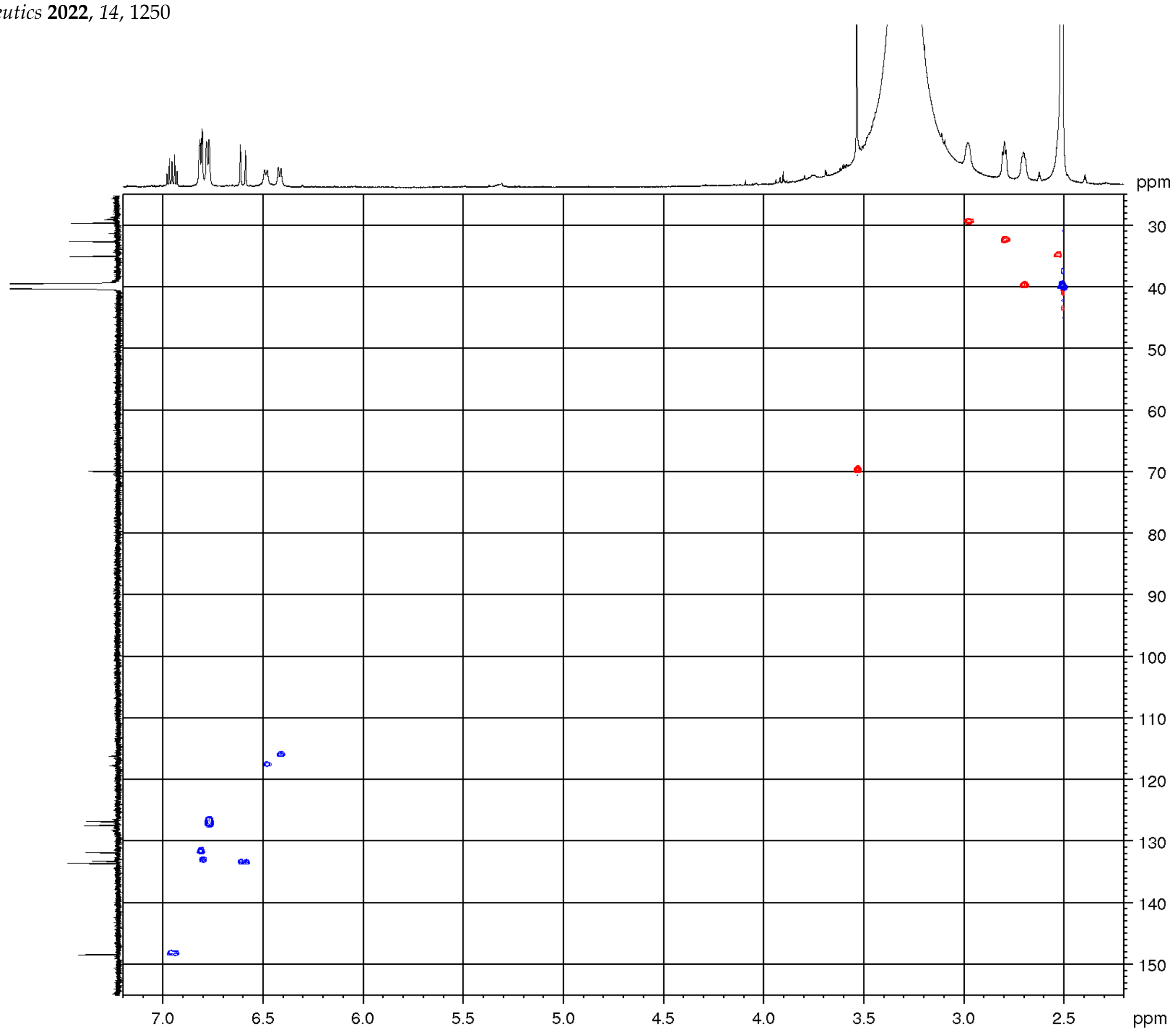

Figure S26. HSQC spectrum of alnusone (**7**) (DMSO-*d*$_6$, 333 K).

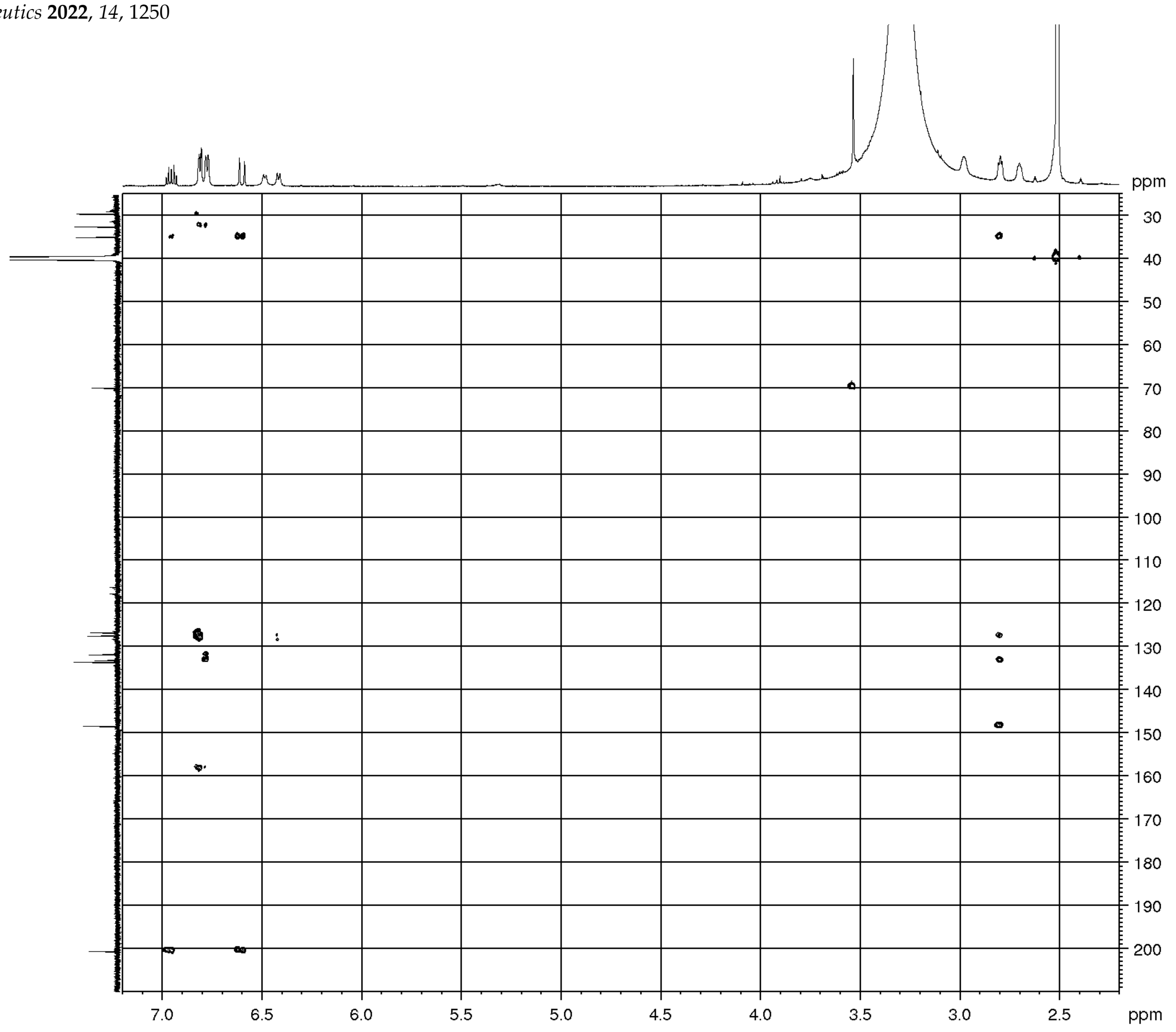

Figure S27. HMBC spectrum of alnusone (**7**) (DMSO-d_6, 333 K).

NMR data of compound **8** (giffonin F): ^{1}H NMR (methanol-d_4, 600 MHz, 295 K): δ 7.48 (dd, $^3J_{H,H}$=8.2 Hz, $^4J_{H,H}$=2.0 Hz, 1H, H-15), 7.16 (dd, $^3J_{H,H}$=8.2 Hz, $^4J_{H,H}$=2.0 Hz, 1H, H-16), 7.08 (dd, $^3J_{H,H}$=8.2 Hz, $^4J_{H,H}$=2.0 Hz, 1H, H-19), 6.94 (dd, $^3J_{H,H}$=8.2 Hz, $^4J_{H,H}$=2.0 Hz, 1H, H-18), 6.41 (d, $^3J_{H,H}$=12.1 Hz, 1H, H-7), 5.29 (dd, $^3J_{H,H}$=12.1 Hz, $^3J_{H,H}$=9.1 Hz, 1H, H-8), 4.84 (s, 1H, H-6), 4.29 (t, $^3J_{H,H}$=9.8 Hz, 1H, H-9), 3.99 (s, 3H, OCH_3-2), 3.67 (s, 3H, OCH_3-4), 3.06 (m, 1H, H-13a), 2.99 (td, $^2J_{H,H}$=12.5 Hz, $^3J_{H,H}$=5.4 Hz, 1H, H-13b), 2.81 (td, $^2J_{H,H}$=12.5 Hz, $^3J_{H,H}$=5.4 Hz, 1H, H-12a), 2.48 (m, 1H, H-12b), 2.45 (m, 1H, H-10a), 1.72 (m, 1H, H-10b). ^{13}C NMR (methanol-d_4, 150 MHz, 295 K): δ 215.6 (C-11), 156.6 (C-17), 152.1 (C-1), 145.2 (C-3), 142.2 (C-4), 138.8 (C-14), 138.3 (C-2), 134.0 (C-19), 133.2 (C-8), 130.7 (C-15), 125.3 (C-5), 125.1 (C-16), 124.9 (C-7), 124.0 (C-18), 108.5 (C-6), 65.3 (C-9), 61.6 (OCH_3-2), 61.5 (OCH_3-4), 51.7 (C-10), 45.2 (C-12), 33.2 (C-13).

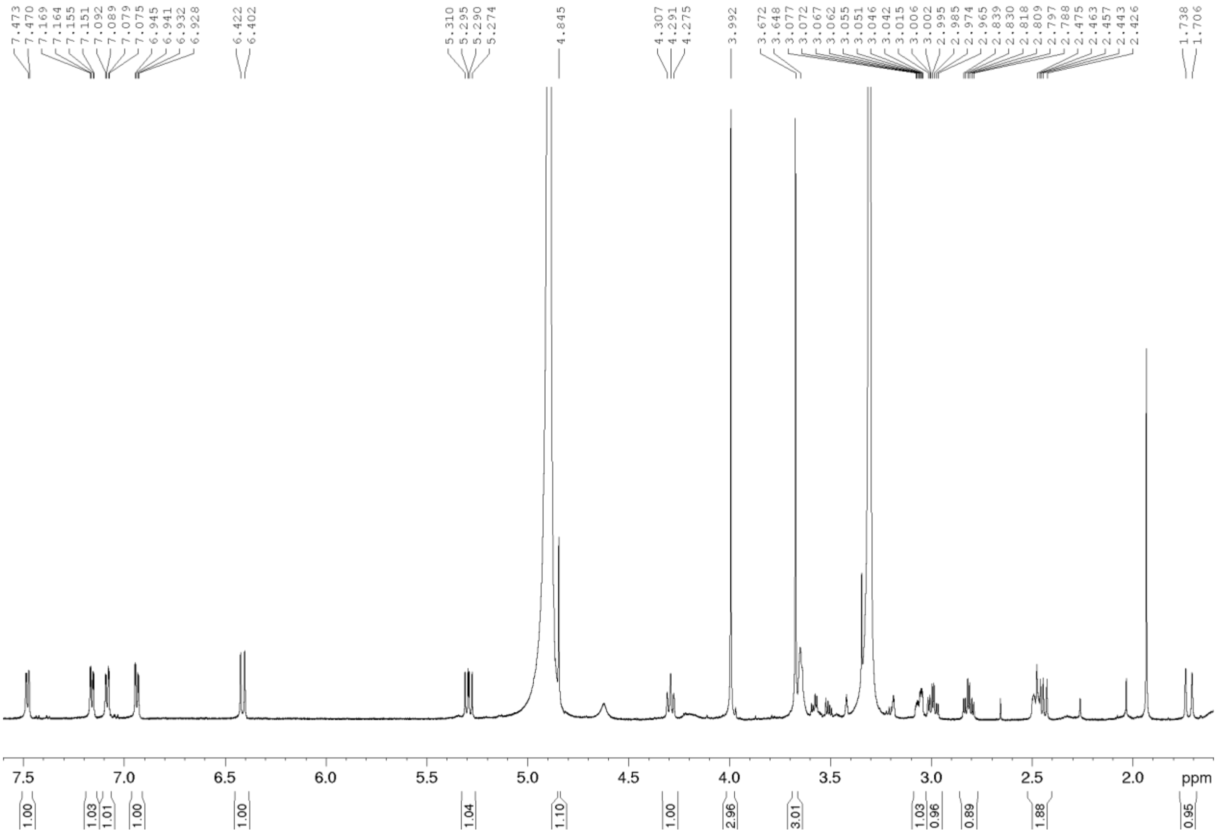

Figure S28. ^{1}H NMR spectrum of giffonin F (**8**) (methanol-d_4, 295 K).

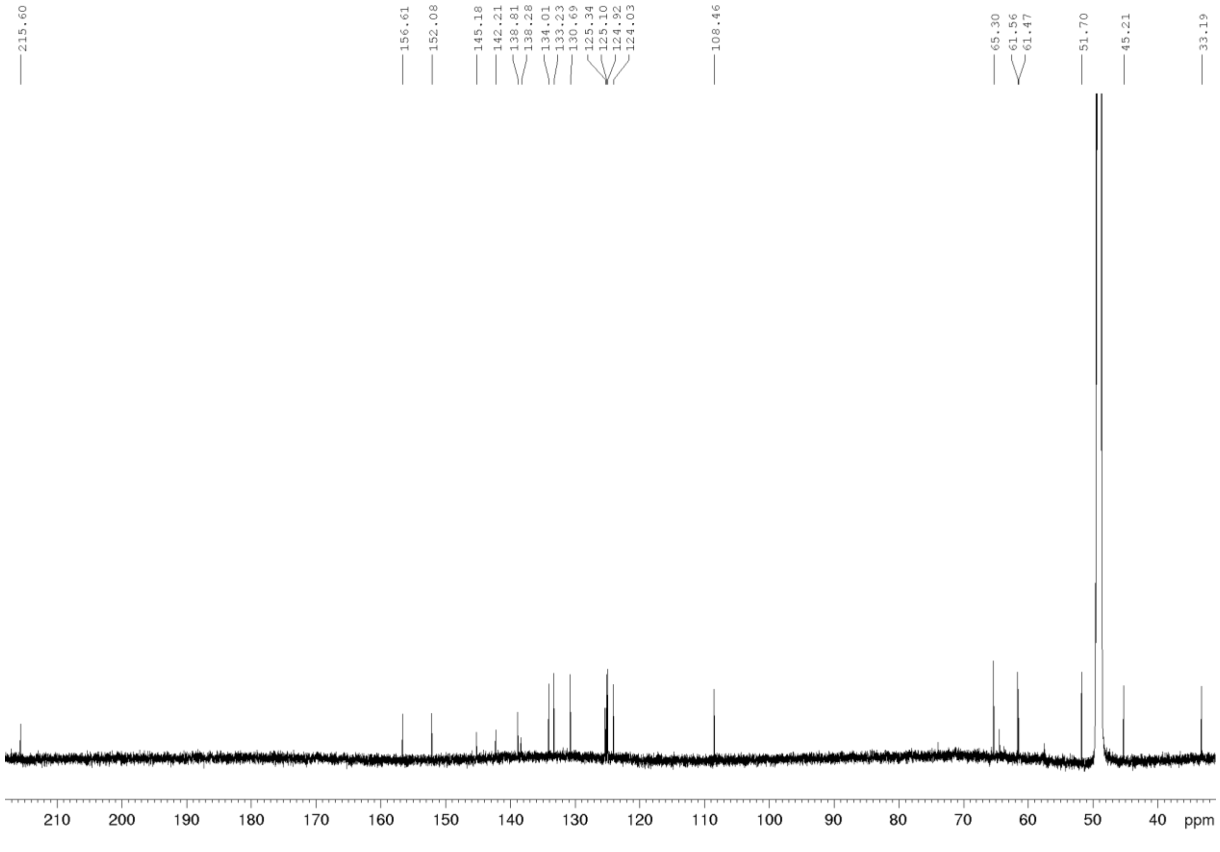

Figure S29. ^{13}C NMR spectrum of giffonin F (**8**) (methanol-d_4, 295 K).

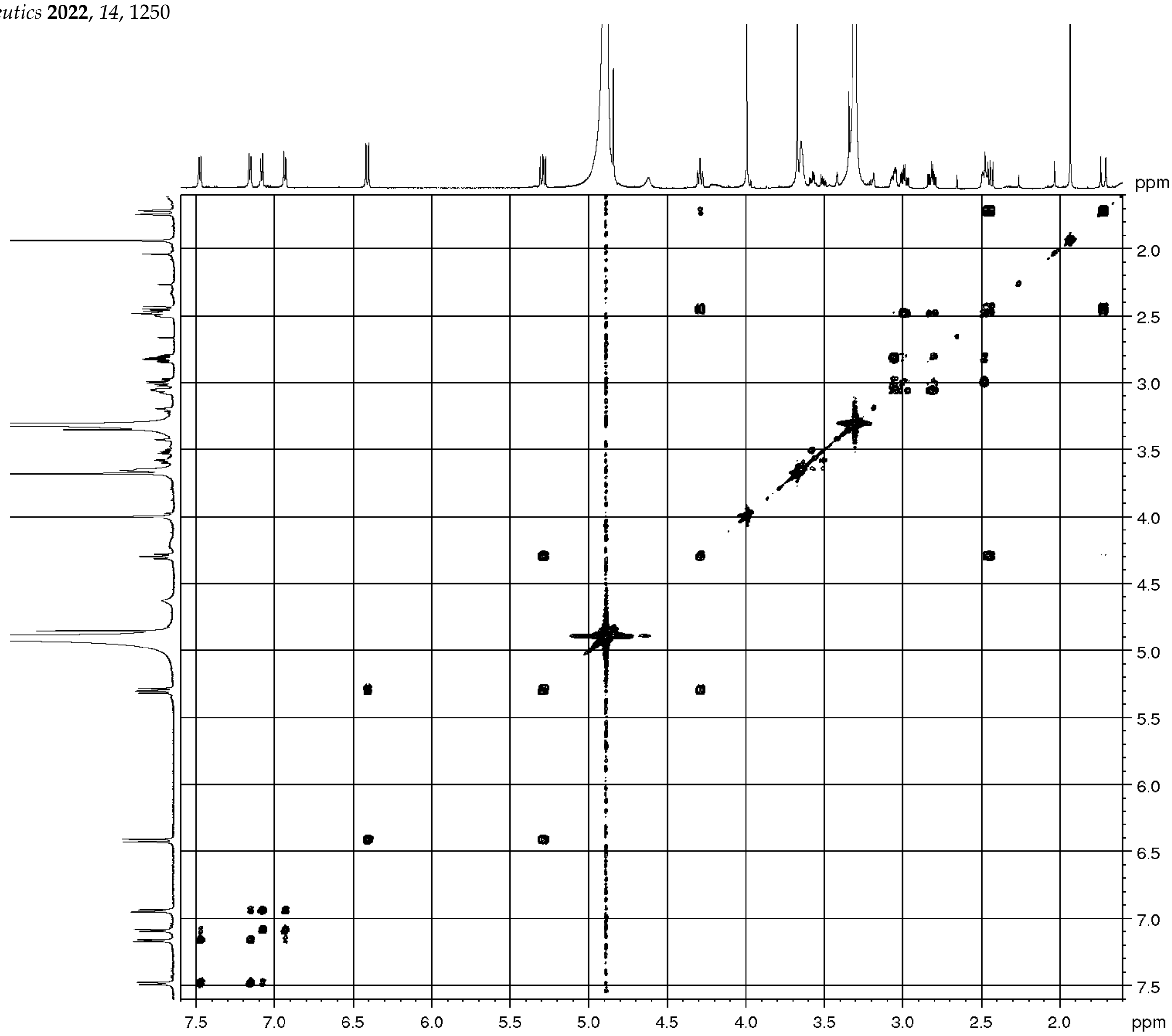

Figure S30. COSY spectrum of giffonin F (**8**) (methanol-d_4, 295 K).

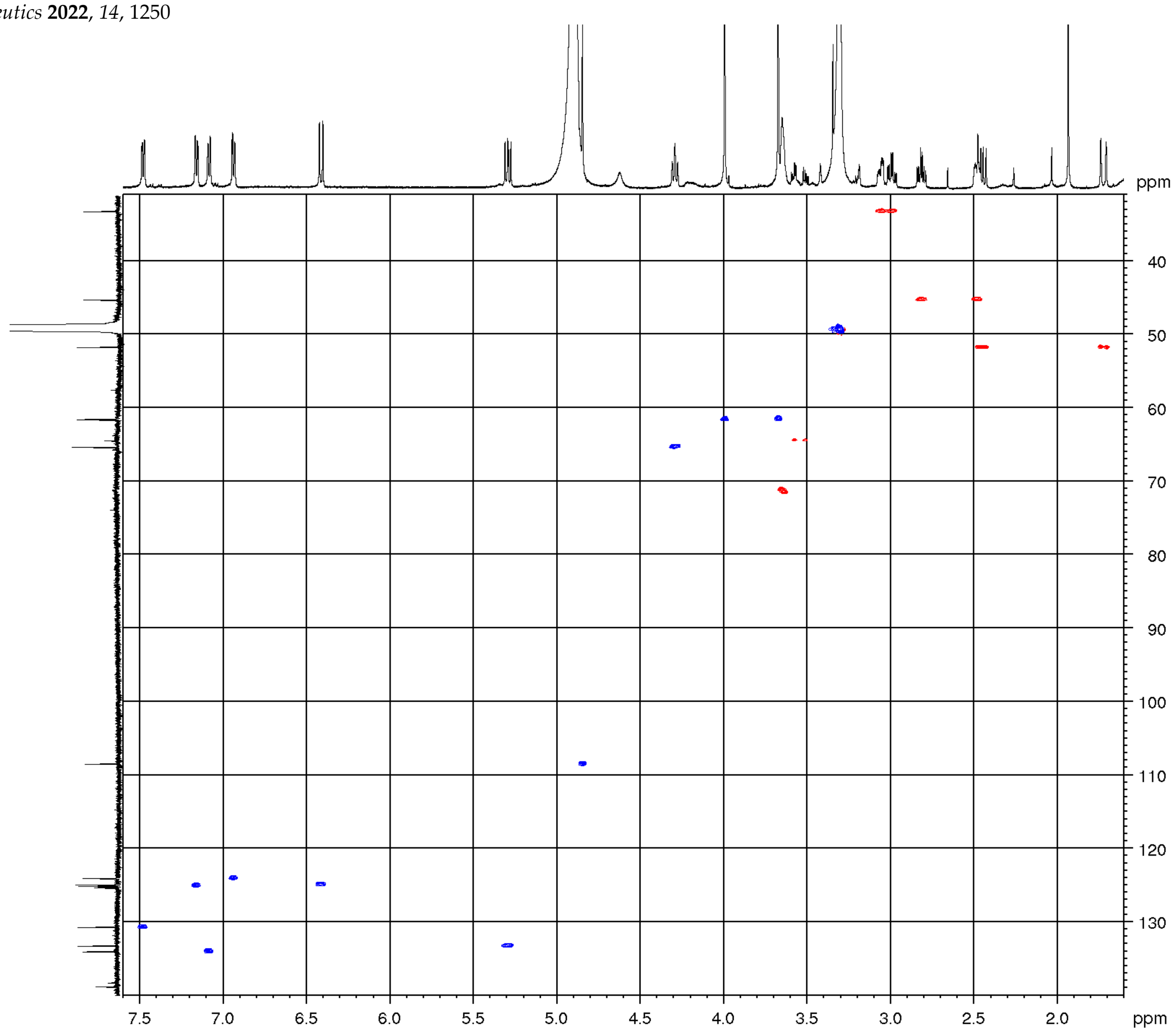

Figure S31. HSQC spectrum of giffonin F (**8**) (methanol-*d*₄, 295 K).

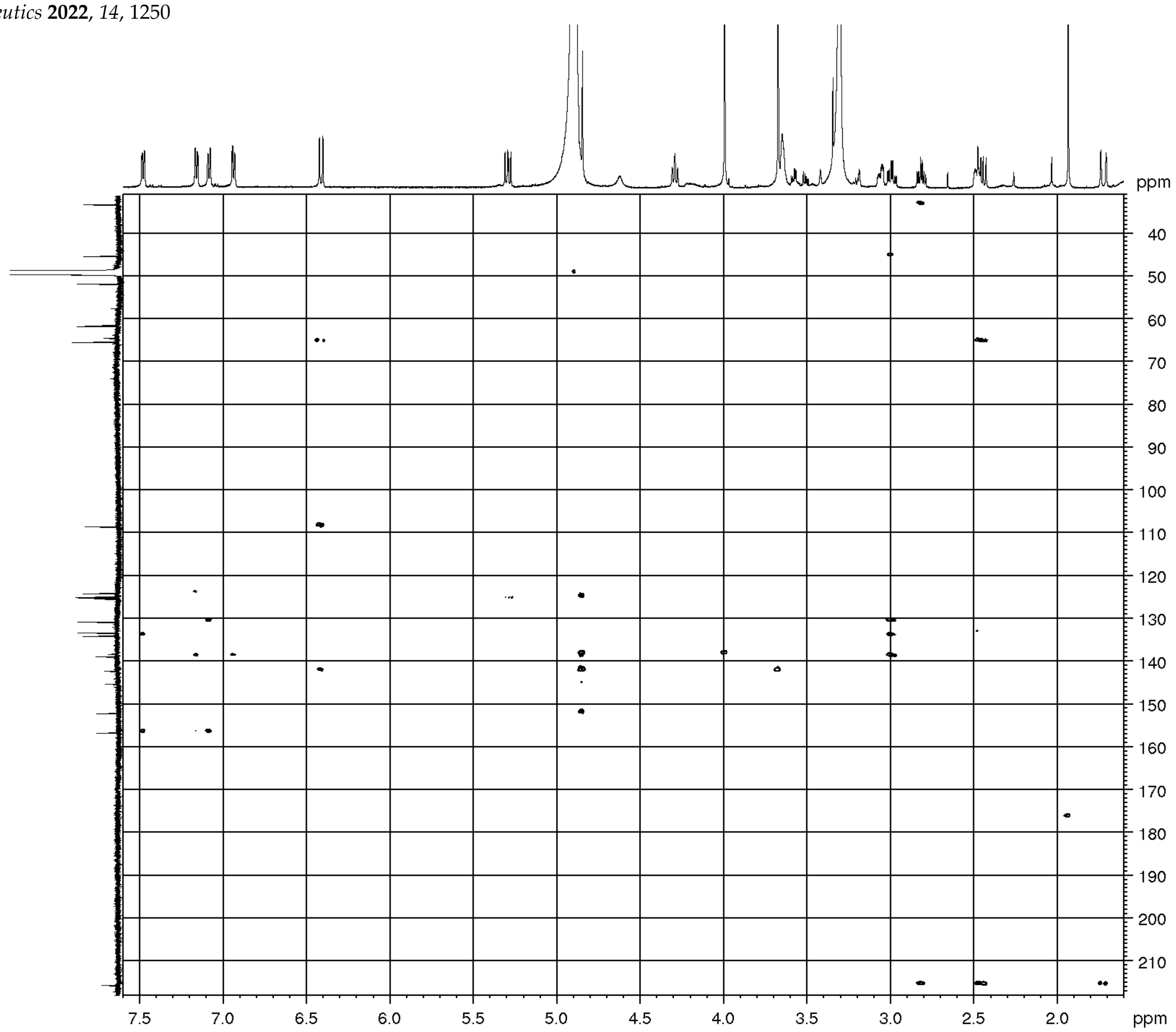

Figure S32. HMBC spectrum of giffonin F (**8**) (methanol-d_4, 295 K).

NMR data of compound **9** (carpinontriol B): ^{1}H NMR (methanol-d_4, 600 MHz, 298 K): δ 7.07 (dd, $^3J_{H,H}$=8.2 Hz, $^4J_{H,H}$=1.9 Hz, 1H, H-5), 7.00 (dd, $^3J_{H,H}$=8.2 Hz, $^4J_{H,H}$=1.9 Hz, 1H, H-15), 6.80 (d, $^3J_{H,H}$=8.2 Hz, 1H, H-4), 6.77 (d, $^3J_{H,H}$=8.2 Hz, 1H, H-16), 6.65 (bd, $^4J_{H,H}$=1.9 Hz, 1H, H-18), 6.36 (bd, $^4J_{H,H}$=1.9 Hz, 1H, H-19), 4.71 (dd, $^3J_{H,H}$=11.9 Hz, $^3J_{H,H}$=4.4 Hz, 1H, H-12), 4.22 (d, $^3J_{H,H}$=10.1 Hz, 1H, H-10), 3.87 (d, $^3J_{H,H}$=10.1 Hz, 1H, H-11), 3.51 (ddd, $^2J_{H,H}$=20.0 Hz, $^3J_{H,H}$=12.5 Hz, $^3J_{H,H}$=2.0 Hz, 1H, H-8a), 3.14 (m, 1H, H-7a), 3.05 (dd, $^2J_{H,H}$=16.0 Hz, $^3J_{H,H}$=4.3 Hz, 1H, H-13a), 2.93 (m, 1H, H-8b), 2.89 (m, 1H, H-13b), 2.84 (m, 1H, H-7b). ^{13}C NMR (methanol-d_4, 150 MHz, 298 K): δ 215.6 (C-9), 153.0 (C-17), 152.1 (C-3), 135.1 (C-19), 135.0 (C-18), 131.4 (C-6), 130.7 (C-14), 130.7 (C-15), 129.5 (C-5), 127.6 (C-1), 126.7 (C-2), 117.0 (C-4, C-16), 78.8 (C-10), 69.8 (C-11), 68.7 (C-12), 37.6 (C-8), 37.1 (C-13), 25.3 (C-7).

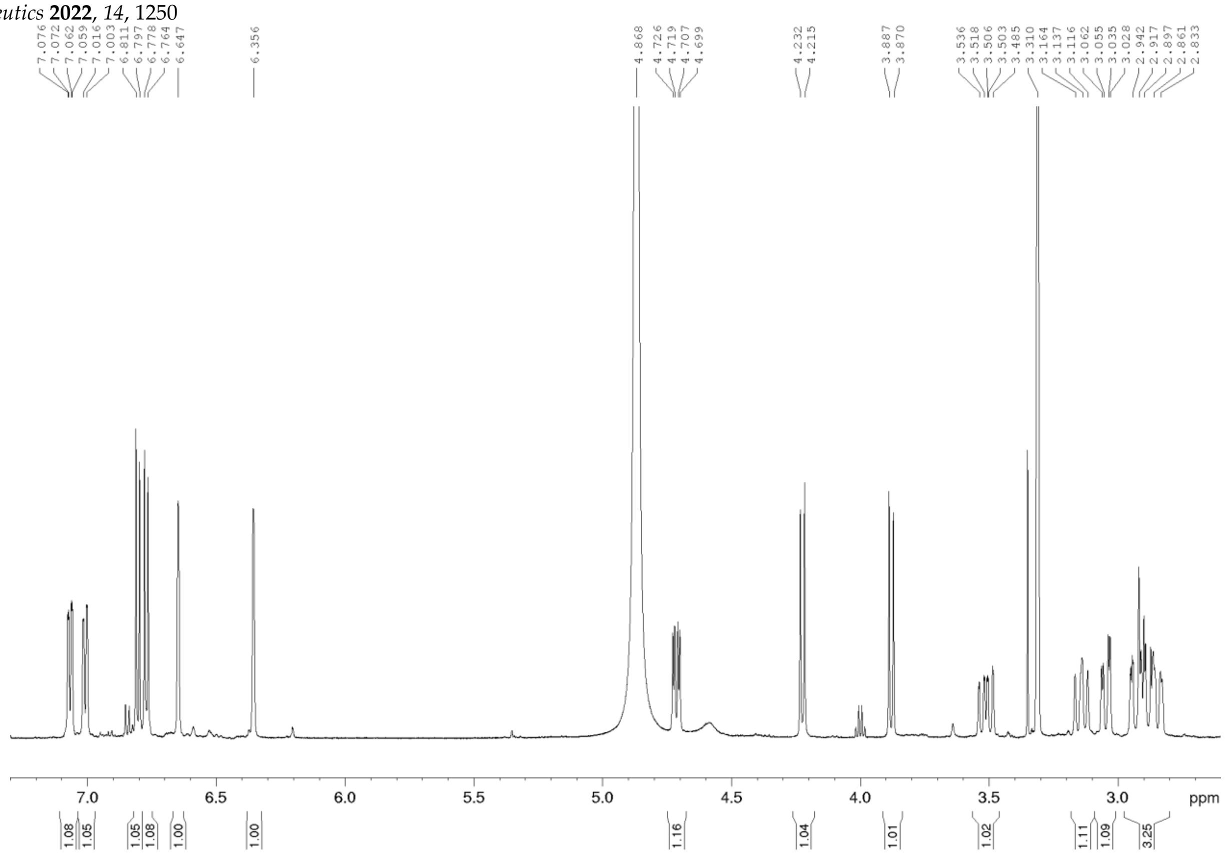

Figure S33. ^{1}H NMR spectrum of carpinontriol B (**9**) (methanol-d_4, 298 K).

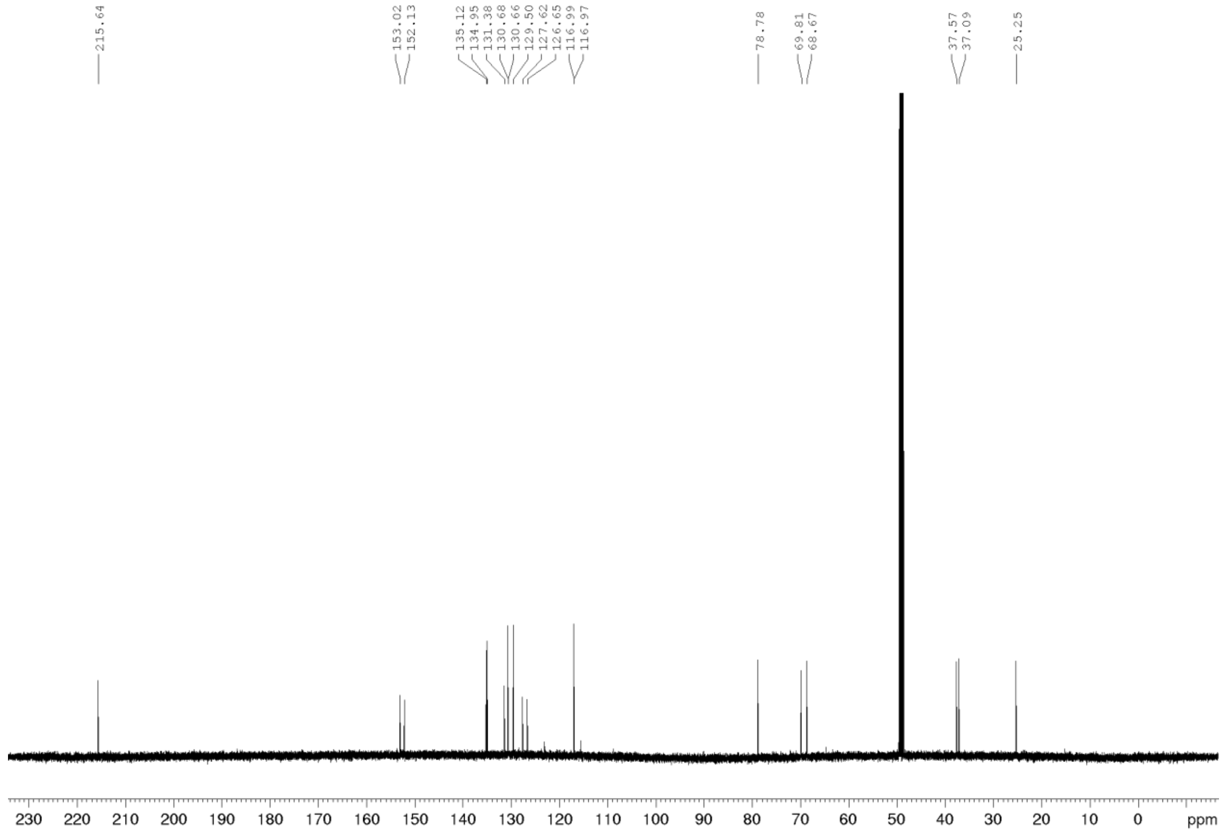

Figure S34. ^{13}C NMR spectrum of carpinontriol B (**9**) (methanol-d_4, 298 K).

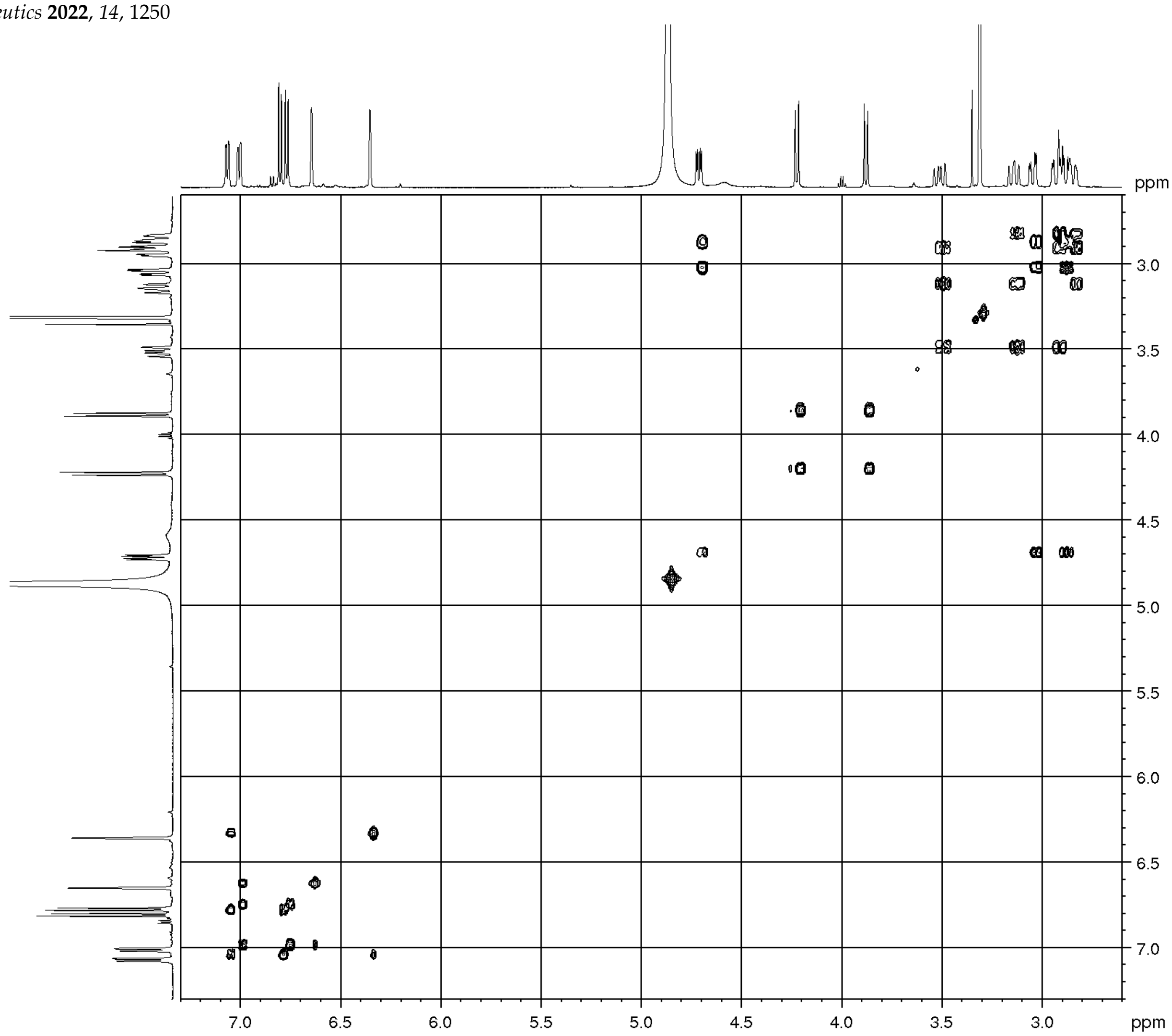

Figure S35. COSY spectrum of carpinontriol B (**9**) (methanol-d_4, 298 K).

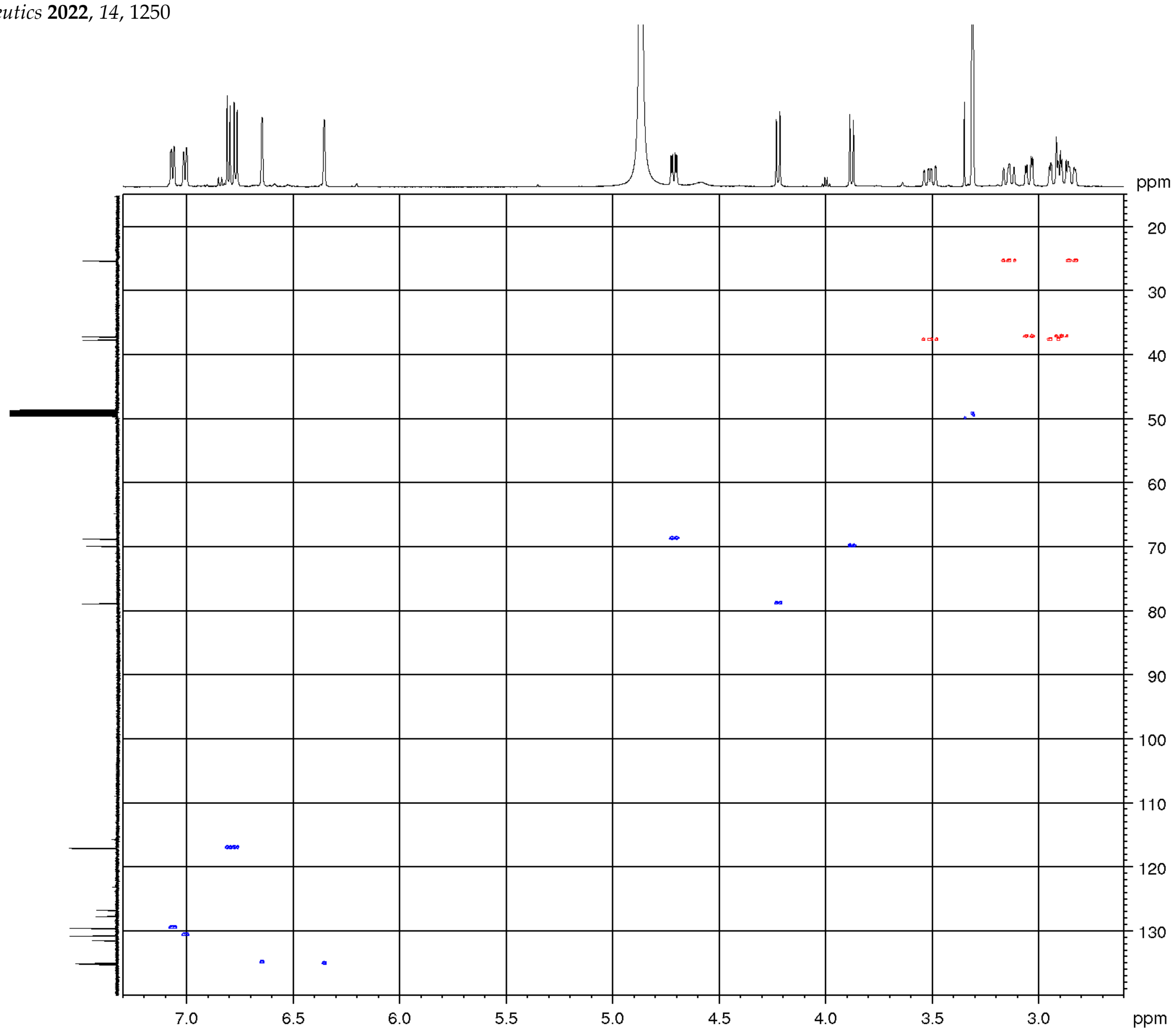

Figure S36. HSQC spectrum of carpinontriol B (**9**) (methanol-d_4, 298 K).

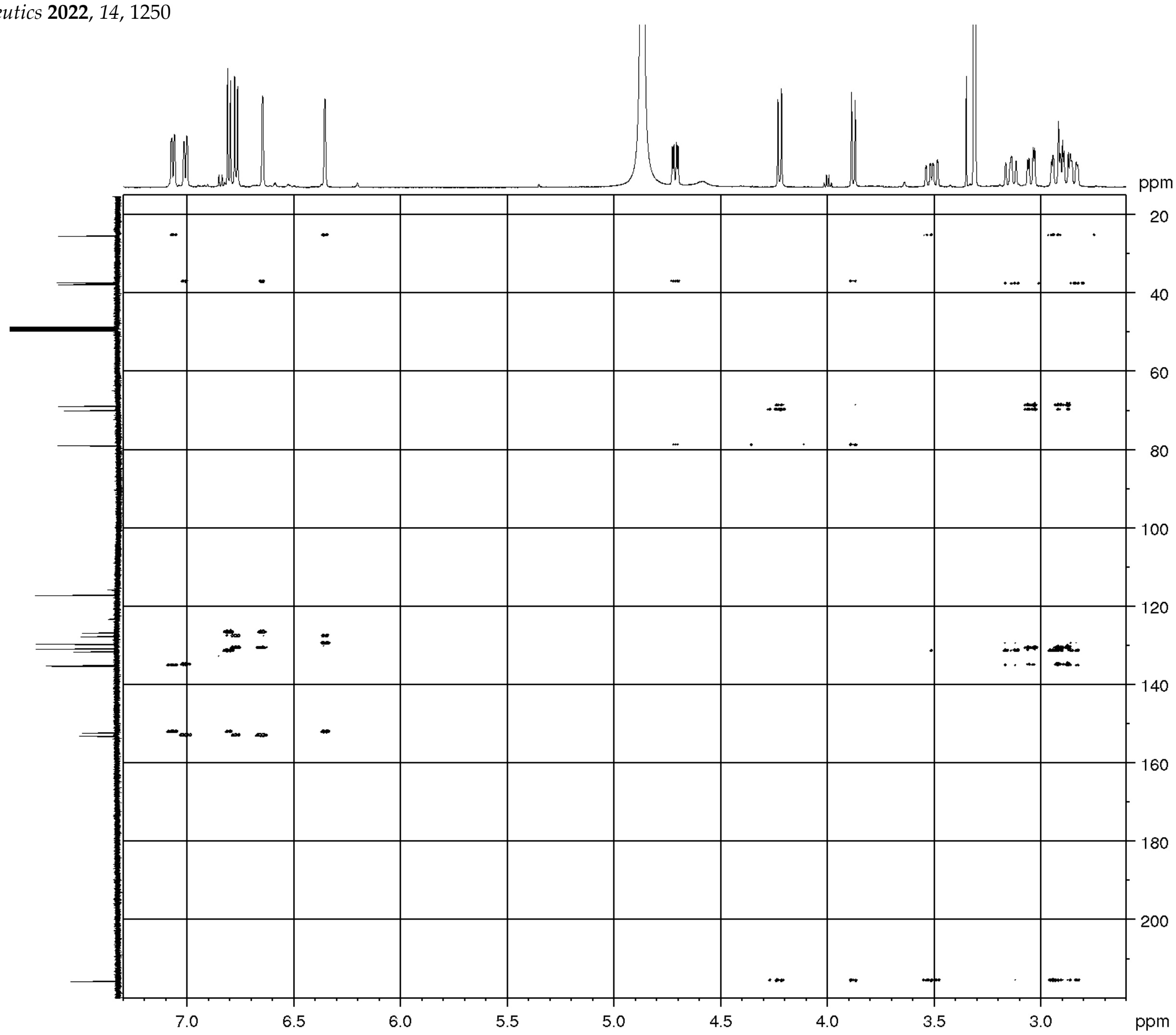

Figure S37. HMBC spectrum of carpinontriol B (**9**) (methanol-d_4, 298 K).
